# Biomass traits and candidate genes for bioenergy revealed through association genetics in coppiced European *Populus nigra* (L.)

**DOI:** 10.1186/s13068-016-0603-1

**Published:** 2016-09-08

**Authors:** Mike Robert Allwright, Adrienne Payne, Giovanni Emiliani, Suzanne Milner, Maud Viger, Franchesca Rouse, Joost J. B. Keurentjes, Aurélie Bérard, Henning Wildhagen, Patricia Faivre-Rampant, Andrea Polle, Michele Morgante, Gail Taylor

**Affiliations:** 1 Centre for Biological Sciences, Life Sciences Building, University of Southampton, Southampton, SO17 1BJ UK; 2 CNR-IVALSA, Sesto Fiorentino, via Madonna del Piano, 10, 50019 Sesto Fiorentino, FI Italy; 3 Laboratory of Genetics, Wageningen University and Research, 6708PB Wageningen, The Netherlands; 4 US1279 EPGV, CEA-IG/CNG, INRA, 91057 Evry, France; 5 Georg-August-Universität Göttingen, 37077 Göttingen, Germany; 6 Dipartimento di Scienze agroalimentari, ambientali e animali, Università di Udine, Via delle Scienze 206, 33100 Udine, Italy; 7 Istituto di Genomica Applicata (IGA), via J. Linussio 51, 33100 Udine, Italy

**Keywords:** Short rotation coppice (SRC), Yield, Lignocellulosic, Genetics, Salicaceae, Leaf area

## Abstract

**Background:**

Second generation (2G) bioenergy from lignocellulosic feedstocks has the potential to develop as a sustainable source of renewable energy; however, significant hurdles still remain for large-scale commercialisation. *Populus* is considered as a promising 2G feedstock and understanding the genetic basis of biomass yield and feedstock quality are a research priority in this model tree species.

**Results:**

We report the first coppiced biomass study for 714 members of a wide population of European black poplar (*Populus nigra* L.), a native European tree, selected from 20 river populations ranging in latitude and longitude between 40.5 and 52.1°N and 1.0 and 16.4°E, respectively. When grown at a single site in southern UK, significant Site of Origin (SO) effects were seen for 14 of the 15 directly measured or derived traits including biomass yield, leaf area and stomatal index. There was significant correlation (*p* < 0.001) between biomass yield traits over 3 years of harvest which identified leaf size and cell production as strong predictors of biomass yield. A 12 K Illumina genotyping array (constructed from 10,331 SNPs in 14 QTL regions and 4648 genes) highlighted significant population genetic structure with pairwise F_ST_ showing strong differentiation (*p* < 0.001) between the Spanish and Italian subpopulations. Robust associations reaching genome-wide significance are reported for main stem height and cell number per leaf; two traits tightly linked to biomass yield. These genotyping and phenotypic data were also used to show the presence of significant isolation by distance (IBD) and isolation by adaption (IBA) within this population.

**Conclusions:**

The three associations identified reaching genome-wide significance at *p* < 0.05 include a transcription factor; a putative stress response gene and a gene of unknown function. None of them have been previously linked to bioenergy yield; were shown to be differentially expressed in a panel of three selected genotypes from the collection and represent exciting, novel candidates for further study in a bioenergy tree native to Europe and Euro-Asia. A further 26 markers (22 genes) were found to reach putative significance and are also of interest for biomass yield, leaf area, epidermal cell expansion and stomatal patterning. This research on European *P. nigra* provides an important foundation for the development of commercial native trees for bioenergy and for advanced, molecular breeding in these species.

**Electronic supplementary material:**

The online version of this article (doi:10.1186/s13068-016-0603-1) contains supplementary material, which is available to authorized users.

## Background

Short rotation coppice (SRC) or short rotation forestry (SRF) *Populus* is widely considered as a promising lignocellulosic feedstock for second generation biofuel production [1, 2]; being fast growing [3], widespread in the northern hemisphere [4], genetically diverse [5, 6], readily transformed [7] and already established as a model tree species [8, 9]. Mapping pedigrees and genetic linkage maps [10–13] exist for a number of *Populus* species [14] and the *P. trichocarpa* genome, which at around 550 Mb is small for a forest tree [15], has been fully sequenced [16]. A number of bioinformatics tools assist in the exploration and utilisation of these genetic and genomic resources; including PopGenIE [17] and POParray [18]. Most recently *Populus* became the first forest tree for which CRISPR/Cas genome editing has been successfully demonstrated [19]. This offers significant potential and suggests that candidate genes identified for traits of interest could be progressed rapidly to commercialisation using such accelerated molecular breeding approaches [14].

Considerable research effort has been employed to elucidate the genetic basis of phenotypes of interest in *Populus* with much focus on mapping quantitative trait loci (QTL) for cell wall composition [20]; biomass yield [1, 21, 22]; biomass distribution [23]; drought tolerance [24, 25]; water-use efficiency (WUE) [26]; pest resistance [13]; bud set and flush [27, 28] and responses to nitrogen deficiency [29], elevated CO_2_ [30, 31] and ozone [32]. Recently, however, inbred mapping pedigrees, which are limited in their recombination events and QTL size in out-breeding populations such as *Populus*, have been replaced with wide natural populations that are particularly beneficial for trees since they capture increased genetic variation [33]. This includes the mapping population utilised in this work; with genotypes drawn from across the western European range for this native tree. Research in this genetic background is particularly important given the tendency for *Populus* commercialisation to be focussed on F_1_ hybrids originating outside Europe [14] and because climate change will require more resilient germplasm planting that will only emerge from a better understanding of the genetic basis of adaptive traits such as biomass production [34].

Association mapping is a powerful technique for elucidating the genetic basis of qualitative and quantitative traits in species of interest, seeking statistical associations between genotypic markers (generally single nucleotide polymorphisms, SNPs) and defined phenotypic qualities within a population [33]. Such associations exist as a result of linkage disequilibrium (LD), defined as the non-random association of alleles at different loci, by which the genotype present at one locus is not independent of another locus [34–36]. LD can result from genetic linkage (i.e. a close physical genomic association reducing or eliminating recombination between two polymorphisms during meiotic division); selection (natural or artificial) and admixture; all of which perturb linkage equilibrium [35, 37]. LD underpins all association genetics studies and can allow the identification or confirmation of candidate genes contributing to the phenotype in question and provide genetic markers to assist in selective breeding efforts [38]. LD in this population has been previously shown to decay rapidly with the value of *r* dropping to half its maximum value within 4 kb [39].

Whilst association mapping can be performed within targeted areas of a genome; for example within candidate genes [40]; falling costs and rapid progress in sequencing and genotyping methods [41, 42] have increased the prevalence of genome-wide association studies (GWAS). Next generation sequencing (NGS) techniques allow large numbers of single nucleotide polymorphisms (SNPs) to be identified within a genome and high-throughput SNP arrays (‘chips’) allow many individuals to be genotyped for multiple markers simultaneously [43]. A number of recent publications in *P. trichocarpa* have made use of a 34 K array covering 3543 genes [44] in GWAS for wood quality [45], biomass, ecophysiology, phenology [46, 47] and disease resistance [48] traits. This array has also been employed in understanding the impact of geographical and environmental factors on phenotypic variation and genetic structure within *P. trichocarpa* across its North American range [49].

Studies in *P.trichocarpa* exceed those published in any other *Populus* species, however, in Europe, *Populus nigra* L. (black poplar) is the native cottonwood (*Aigeiros*); also found across North Africa and Central Asia [50, 51]. It is an ecologically important and endangered riparian, pioneer species [52–54]; for which only extremely small-scale candidate gene association studies have previously been reported. For example, Guerra et al. [55] used 433 SNPs from 39 candidate genes for cellulose and lignin biosynthesis to genotype an association population of 599 individuals; identifying 6 trait-marker associations. It follows that prior to the development of the genotyping array utilised here [39] the study of population structure within its European range had been restricted to analysis of small numbers of AFLP and microsatellite markers [53, 54]. Understanding population structure is an important consideration for conducting GWAS [56] and providing robust trait-associated markers for subsequent advanced breeding programmes [57, 58]; as well as being of value for conservation efforts in threatened species such as this one [54, 59, 60].

The aim of this research was to elucidate the links between biomass traits and their underlying genetic architecture. In particular we aim to unravel complex traits considered important for the development of the native black poplar as a sustainable source of lignocellulosics for the bioenergy industry, particularly across Europe where native species are likely to be preferred. This work describes the first use of phenotyping and genotyping data together from a 12K Illumina Infinium genotyping array which provides SNP markers an order of magnitude greater in number than previous studies in *P. nigra* and with coverage of a far greater proportion of the genome. We focus on traits with moderate to high heritability and considered to be underpinning biomass production including leaf development, stomatal patterning, height and stem volume index [31, 61] as well as saccharification potential [62]. These genetic and phenotypic datasets have been considered together in the first GWAS study in this species, identifying candidate genes for bioenergy traits as well as valuable insight into the challenges and opportunities for further such studies in both this and other significantly structured and geographically disparate populations.

## Methods

### Mapping population and UK field trial

**Fig. 1:**
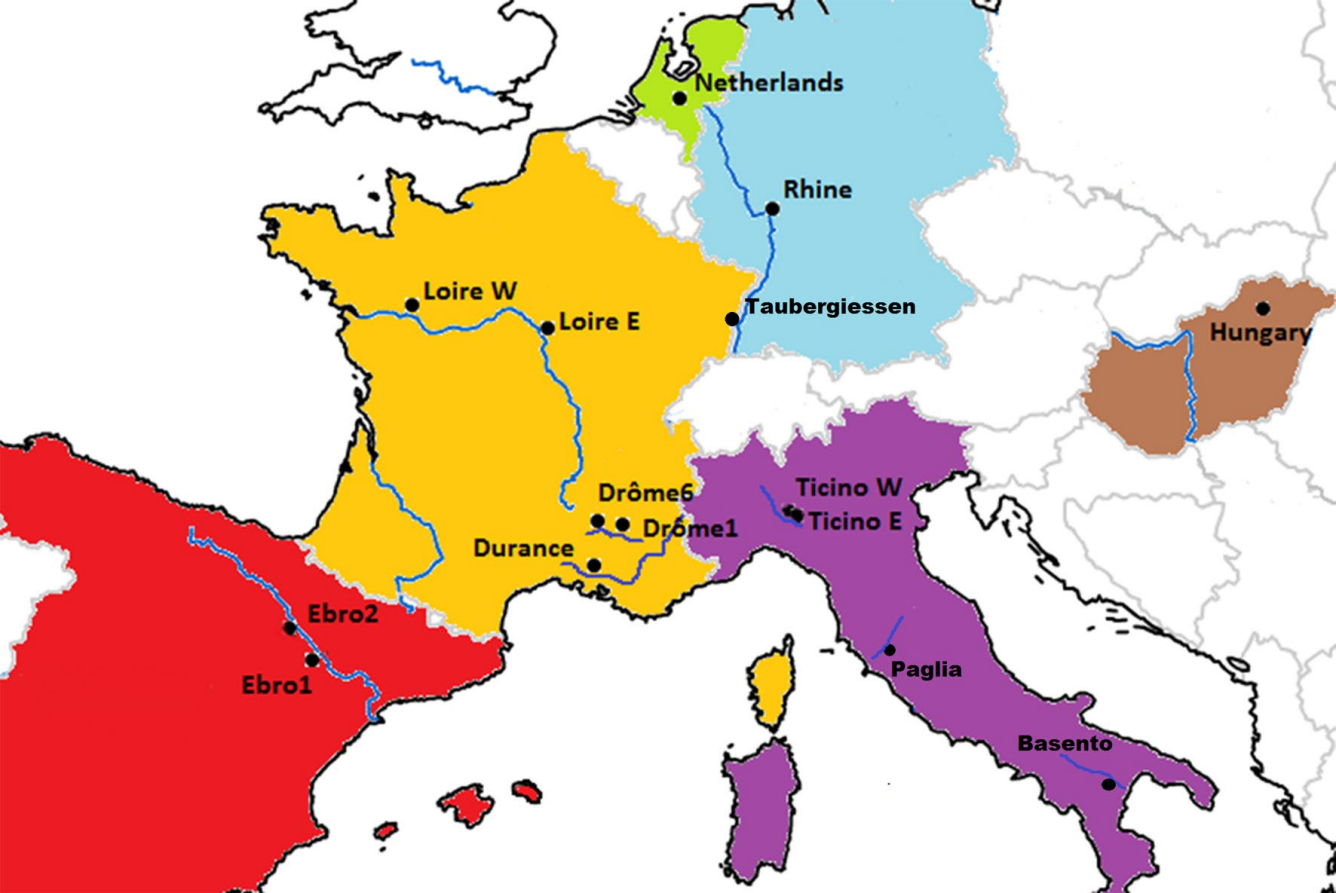
Map illustrating the nations and major river locations from which the *P. nigra* association population is drawn and the colours employed to illustrate these nations in subsequent figures

The *P. nigra* population [54, 63, 64] is a wide, natural population of more than 1000 diverse genotypes drawn from riparian ecosystems across Western Europe; namely France, Italy, Spain, Germany, Netherlands and Hungary [39, 65]. Cuttings taken from mature trees in situ were established and propagated in a stool bed at INRA, UAGPF, Orléans and ramets from this stool bed, established for more than 5 years, were cut and established for this work in a field trial (common garden) in Northington, south-east UK; (51°12′N, 1°21′E) in 2009. It is possible that sites of propagation can significantly influence structural and functional aspects of the genome in clonally propagated *Populus*, including response to drought stress [66]. In this study, however, sourcing all plant material for this trial from a stable, well established stool bed should act to minimise this variation, although it cannot be entirely ruled out. Such effects may otherwise bias estimates of heritability, inter-trait correlations and genetic potential in common garden experiments [67]. 931 genotypes (714 genotyped on the Illumina array representing 20 sampled sites) were planted at 0.80 × 0.80 m spacing in double rows, spaced by 3 m. The site was laid out in six fully replicated, randomised blocks with 4 rows per block and a double row of guards surrounding the site as a whole. Trees were coppiced to 5 cm in February 2010 and 2013 and received mechanical weed control as required. No fertiliser was applied at any time or irrigation post establishment, although trees were irrigated in 2009. The latitude and longitude of the sampled subpopulations and their sample sizes (*n*) are provided in Table 1; a map of the region from which the population is drawn is shown in Fig. 1.

**Table 1:** Sites of Origin (SO) for *P. nigra* association mapping population at Northington, UK

SO	Nation	*N*	Latitude°N	Longitude°E
Basento	Italy	16	40.5	16.4
Paglia	Italy	21	42.8	11.8
Ticino-North	Italy	56	45.3	9.0
Ticino-South	Italy	37	45.2	9.1
Bonny	France	33	47.6	2.8
Dranse	France	35	46.4	6.5
Drome 1	France	55	44.7	5.4
Drome 6	France	53	44.8	4.9
Erstein	France	13	48.4	7.7
Guilly	France	31	47.8	2.3
Ramieres	France	37	44.7	4.9
Rhinau	France	19	48.3	7.7
Loire	France	44	46.4	3.2
Strasbourg	France	18	48.6	7.8
Taubergiessen	France	4	48.3	7.7
Val Allier	France	134	46.4	3.3
Ebro-Alfranca	Spain	24	41.6	1.0
Ebro-Novillas	Spain	24	41.9	1.4
Kuhkopf	Germany	33	49.8	8.5
Netherlands	Netherlands	23	52.1	5.7
Individuals	France (2), Italy (1), Hungary (1)	4	–	–

Subpopulation names are given in the first column followed by the country within which they are located. The number of individual genotypes within each subpopulation is provided (*N*) and their mean latitudinal and longitudinal coordinates for statistical analysis and calculation of pairwise geographic distances. “Individuals” are unique genotypes from outside of the given subpopulations

### Phenotyping for bioenergy-related traits

As shown in Additional file 1: Figure S1, in February 2011 (1st year of growth post 1st coppice), February 2012 (2nd year of growth post 1st coppice) and November 2013 (1st year of growth post 2nd coppice) leading stem height and all primary stem diameters (22 cm above the ground) were measured for all trees for all genotypes and used to calculate stem-volume index (SVI) as a proxy to biomass yield [68] according to the equations:

$${\text{Area of individual stem}}\,\left( {A_{n} } \right)\left( {{\text{mm}}^{2} } \right) = \left( {{D \mathord{\left/ {\vphantom {D 2}} \right. \kern-0pt} 2}} \right)^{2} * \pi$$

$${\text{Total basal area }}\left( {\text{BA}} \right) = \sum {\left( {A_{1} ,A_{2} , \ldots A_{n} } \right)}$$

$${\text{SVI}}\left( {{\text{cm}}^{3} } \right) = {\text{BA}} * \text{H}$$

where H is the height of the leading stem and n is the number of primary stems (i.e. all stems which originate from the original main stem). Additionally, following the 2013 measurements 50 trees were cut, oven-dried for 48 h at 105 °C and weighed to allow estimated oven-dry biomass (EB) to be calculated from SVI (see Additional file 1: Figure S2). In August 2012 (3rd year of growth post 1st coppice) main stems from each tree were sampled at 1 m above the ground and assayed for wood saccharification potential (SP) according to the methodology described by Van Acker et al. [69]. In brief debarked, air-dried samples were milled in a Retsch 300MM Mixer Miller with the resultant powder sieved and the fraction falling between 150 and 850 µm retained. Moisture content was calculated from weight loss of an aliquot of each sample after oven-drying at 105 °C and desiccation to reach a constant weight. A 10 mg sample of un-dried powder underwent acid pre-treatment and ethanol wash steps followed by 48 h saccharification with fungal cellulose (*Trichoderma reesei*) and cellobiase (*Aspergillus niger*) enzymes (Sigma-Aldrich, USA) at 55 °C in a rotating thermomixer. Supernatant was assayed with GOD-POD (glucose oxidase, horseradish peroxidase and ABTS dye) solution [69, 70] which undergoes a colour change on reaction with glucose through the oxidation of the ABTS dye; thus permitting spectrophotometric (ELx800 Absorbance Reader, BioTek, USA) glucose quantification from sample absorbance at 405 nm. SP is calculated as sample glucose yield as a percentage of post pre-treatment oven-dry weight.

In August 2013 (1st year of growth post 2nd coppice) the first, mature leaf was sampled from the main stem for all genotypes in the course of a single week and imaged. Epidermal cell imprints were taken from the abaxial leaf surface using clear nail varnish and Sellotape^®^ and mounted on glass slides as described previously [30]. Slides were viewed with a Zeiss light microscope and imaged with a mounted digital camera. Image J [71] was used to find mature leaf area (LA) from the scanned leaf images [24, 72] and to find epidermal cell area (CA; calculated as the mean average of ten cells per image) and epidermal cell (ECD) and stomatal densities (SD) from the abaxial imprint images [30, 73]. These were used to calculate stomatal index (SI) according to the equation:

$${\text{SI}}\left( \% \right) = \left[ {{{\text{SD}} \mathord{\left/ {\vphantom {{\text{SD}} {\left( {{\text{SD}} + {\text{ECD}}} \right)}}} \right. \kern-0pt} {\left( {{\text{SD}} + {\text{ECD}}} \right)}}} \right] * 100$$

And epidermal cell number per leaf (CNPL) according to the equation:

$$\text{CNPL} = {{\text{LA}} \mathord{\left/ {\vphantom {{\text{LA}} {\text{CA}}}} \right. \kern-0pt} {\text{CA}}}$$

Additionally, the leaves were oven-dried at 80 °C for 48 h and weighed enabling specific leaf area (SLA) to be calculated according to the equation:

$${\text{SLA}}\left( {{{{\text{mm}}^{ 2} } \mathord{\left/ {\vphantom {{{\text{mm}}^{ 2} } {\text{g}}}} \right. \kern-0pt} {\text{g}}}} \right) = {{\text{LA}} \mathord{\left/ {\vphantom {{\text{LA}} {{\text{Leaf}}\,{\text{mass}}}}} \right. \kern-0pt} {{\text{Leaf}}\,{\text{mass}}}}$$

### Statistical analysis

For each trait only genotypes with measurements for at least two replicates and which had been genotyped on the Illumina array were considered in statistical analyses; this in view of the risk of undetected clonal duplication from nature among so-called ‘unique’ ungenotyped individuals in this species [39, 52, 54]. Traits were tested for normality and transformed as required before a general linear model (GLM) was conducted for each in SPSS’ [74] “univariate” GLM function:

$$Y_{\text{ijk}} = {\mu } + S_{i} + \, G_{\text{j(i)}} + \, B_{k} + \, \varepsilon_{\text{ijk}}$$

where µ is the group mean, S_i_ is the effect of site of origin *i* (SO, see Table 1) considered as fixed and* G*_j(i)_ and B_k_ are the effects of genotype *j* (nested within SO) and block *k*, respectively; both considered as random. In the case of saccharification potential where sample processing was completed in multiple runs over several weeks the factor ‘Run’ was additionally included as a random effect to account for laboratory drift. Individual genotypes (singlet genotypes not sampled from a defined river population) were excluded from this analysis but included in GWAS. In view of the significant block effect (*B*_*k*_) found for all traits (Additional file 3: Table S1) the ‘EMMEANS’ function was employed in SPSS [74] to provide block adjusted estimated marginal means for each genotype for each trait and these were used for all subsequent, downstream analyses. Minitab [75] was used to find Pearson’s correlation coefficient (*r*) for pairwise correlations between traits and with latitude and longitude of origin.

### Genotyping data

The genotyping data utilised in this work arises from a 12 K Illumina Infinium II Genotyping BeadChip array and full details of the DNA extraction, sequencing, design and quality testing for which have been recently reported [39]. In summary, SNPs were called from the re-sequencing and alignment of 51 *P. nigra* genotypes (4 high coverage individuals, >25× and 47 low coverage individuals, 2–21×). SNPs selected for the array were drawn from 14 QTL regions and 2916 candidate genes (based on transcriptome studies and the literature) for biomass yield, bud phenology, wood quality, rust resistance and water-use efficiency traits as well as 1732 additional gene models spread throughout the genome [39]. The population (1106 individuals of which 714 are considered in this work) was genotyped using this array according to Illumina’s Infinium protocol. After Illumina technical dropout 9127 SNPs (88 % of initial 10,331) remained on the array of which 8259 (located within 4903 genes, average of 1.68 SNPs per gene [39]) were polymorphic and showed good quality genotype clustering and signal intensity. A further 593 SNPs were removed as unsuitable for GWAS as follows: no minor allele homozygotes (208); failed heritability-based SNP validation (165); GenTrain score <0.50 (208); SNP not assigned to one of 19 linkage groups (11) and duplicated marker on array (1). The resulting 7666 SNP marker set for the 714 individuals cultivated and phenotyped at the Northington site was filtered in TASSEL [76] to remove markers with minor allele frequency (MAF) <0.05; minimum call rate <0.90 and heterozygote frequency >0.95 to produce a final marker set of 7343 informative SNPs for association analyses (Additional file 2).

### Population Genetic Structure

The 7343 SNP marker set was further filtered for population genetic structure analysis. First, markers were filtered for Hardy–Weinberg equilibrium (HWE) in R using the function ‘HWChisqMat’ in the package ‘Hardy–Weinberg’ [77]. This provided 4029 markers of which 3279 had complete information (no missing data). These markers were then filtered in PLINK [78] for linkage disequilibrium (LD) at *r*^2^ < 0.2 [47, 79] to produce a second, reduced marker set of 2390 putatively neutral, unlinked SNPs for genetic structure analyses.

Genetic structure was investigated by three approaches:

I. The reduced marker set (2390 markers) was entered in the program STRUCTURE [80] which employs model-based clustering for inferring population structure from genotyping marker data. It may be utilised to estimate the value of *K*, i.e. the number of subpopulations or clusters of genotypes within a population and to produce a Q-matrix in which individual genotypes are probabilistically assigned to *K* clusters with the proportional likelihood of membership of a given cluster expressed as a decimal between 0 and 1 and with individual probabilities summing to 1 across all clusters for a given genotype. In this instance STRUCTURE’s admixture model with correlated allele frequencies [81] was used to model *K*’s 1–10 (to ensure the capture of the true value of *K*) with ten iterations for each value of *K* and 20,000 burn-in and 100,000 run-length for each iteration. The ‘Structure Harvester’ tool at UCLA [82] was then used to find the best estimate for the true value of *K* according to the method of Evanno et al. [83].

II. Principal component analysis (PCA) of genetic variance in the R package ‘prcomp’ [84] was performed using both the full (7343 SNPs) and reduced (2390 SNPs) marker sets. The number of significant principal components was determined by a broken stick model [85] implemented in the R package ‘vegan’. The significant principal components from the reduced marker set were employed in genetic structure correction in GWAS model II (see below). The eigenvalue loadings from the PCA of the full marker set were used to identify top loading SNPs (top 0.2 % of eigenvalues, 15 SNPs) for PC1 and 2 with a view to locating chromosomal regions enriched in markers related to population genetic differentiation [49].

III. Pairwise F_ST_ (genetic distance) estimates were calculated between the 20 represented sampled populations in the program Arlequin 3.5 [86]. A PCA was performed on the biomass and leaf trait data (i.e. excluding SP for which the Dranse sub-population was not represented) and Euclidian distances calculated between the 20 subpopulations using the first 2 PCs of the phenotypic variation. Pairwise geographic distances between subpopulations were calculated using the haversine formula [87]. Simple and partial Mantel tests [88–90] were then conducted in Arlequin (1000 permutations) between these three pairwise distance matrices where the correlation coefficient between genetic and geographic distances controlling for phenotypic distance (Gen, Geog|Pheno) is considered a measure of isolation by distance (IBD) and the correlation coefficient between genetic and phenotypic distances controlling for geographic distance (Gen, Pheno|Geog) is considered a measure of isolation by adaption (IBA) [91].

### M_eff_, GWAS, model selection and heritability

Effective marker number (*M*_eff_) in the full 7343 marker set (accounting for non-independence between markers arising from LD) was calculated in the Genetic type 1 Error Calculator (GEC) which provides a robust estimation of the number of independent tests being performed for multiple test correction in GWAS; so as to control the genome-wide type 1 error rate at 0.05 [92]. The genome-wide significance level for trait-marker associations from the models below was then calculated as α = 0.05/*M*_eff_. *M*_eff_ was found to equal 5690 and thus the threshold was calculated as *α* = 8.79 × 10^−6^.

Six distinct models were considered for GWAS and executed in TASSEL [76] for all traits using the full marker set. MLMs (models 4 and 5) were run using optimum compression:

1. Simple general linear model (GLM) without correction for population genetic structure:

$$Y = X{\beta } + e$$

where *Y* is a vector of phenotypic values; β is an unknown vector containing fixed effects for genetic markers; *X* is the known design matrix and e is the unobserved vector of random residuals.

2. GLM using significant PCs from reduced marker set PCA for genetic structure correction (P-model) with notation as for model I but β contains fixed effects for both genetic markers and population structure (PCs).

3. GLM using Q-matrix with optimal *K* from STRUCTURE for genetic structure correction (Q-model) with notation as for model II but population fixed effects in β derived from Q-matrix instead of PCs.

4. Mixed linear model (MLM) with a kinship matrix created from the reduced, 2390 marker set using the Efficient Mixed Model Association (EMMA) algorithm [56] in the *R* package ‘GAPIT’ [93] for genetic structure correction (K-model):

$$Y \, = \, X{\beta } + \, Zu + \, e$$

where *Y* is a vector of phenotypic values; β is an unknown vector containing fixed effects for genetic markers;* u* is an unknown vector of random additive genetic effects; *X* and *Z* are the known design matrices and e is the unobserved vector of random residuals.

5. The full animal model [94] MLM using Q-matrix from STRUCTURE and EMMA kinship matrix for genetic structure correction (Q + K-model) with notation as for model IV but β contains fixed effects for both genetic markers and population structure (Q-matrix).

6. Full animal model MLM using significant PCs of genetic variation and EMMA kinship matrix for genetic structure correction (P + K-model) with notation as for model IV but β contains fixed effects for both genetic markers and population structure (PCs).

To determine the most appropriate of the above models for identifying reliable trait-marker associations on a trait-specific basis the unified mixed model framework used by McKown et al. [47] was employed; utilising the Bayesian Information Criterion (BIC) to compare log-likelihood values between models [95]. This was performed in R using the functions ‘lm’ and ‘lmekin’ in the packages ‘coxme’ [96] and ‘MuMIn’ [97].

The R package ‘heritability’ [98] was employed to calculate *h*^2^ for each phenotype using the individual observations for each genotypic replicate (transformed for normality). The function ‘marker_*h*^2^′ was used to fit a mixed model using the EMMA kinship matrix and the seven significant principle components of the genetic variation as covariants. Narrow sense heritability is calculated according to the equation:

$$h^{2} = {{\sigma^{2} } \mathord{\left/ {\vphantom {{\sigma^{2} } {\left( {\sigma_{g}^{2} + \sigma_{r}^{2} } \right)}}} \right. \kern-0pt} {\left( {\sigma_{g}^{2} + \sigma_{r}^{2} } \right)}}$$

where σ_g_^2^ is the additive genetic variance and σ_r_^2^ is residual (error) variance such that σ_g_^2^ +σ_r_^2^equates to the total model variance. Trait heritabilities were regressed against their absolute correlation coefficients with latitude and longitude in Minitab.

## Results

### Trait variation, correlations and heritabilities

**Fig. 2:**
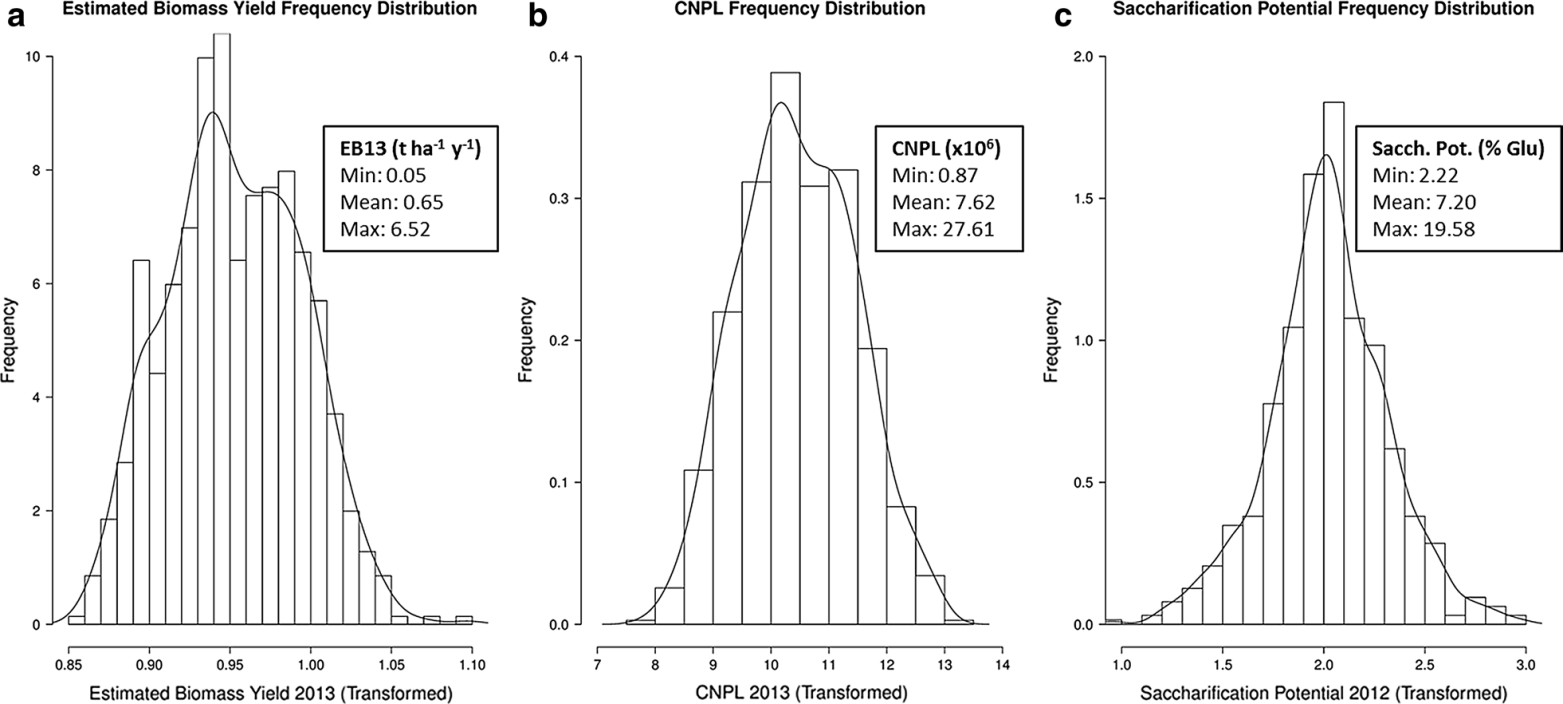
Histograms illustrate trait frequency distribution following Box-Cox transformation for **a** Estimated biomass yield 2013 (EB-13); **b** Epidermal cell number per leaf (CNPL-13) and **c** Saccharification potential (glucose yield) 2012 (SP-12)

**Fig. 3:**
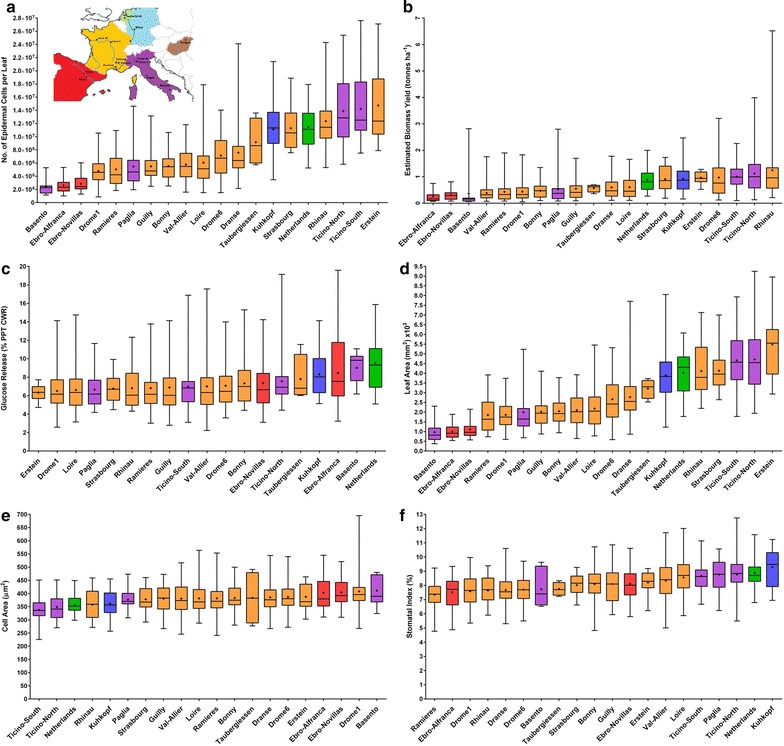
*Box plots* depict range, interquartile range, median and mean (cross) for **a** Epidermal cell number per leaf 2013 (CNPL-13); **b** Estimated oven-dry biomass yield 2013 (EB-13); **c** Saccharification potential (glucose release) 2012 (SP-12); **d** Leaf area 2013 (LA-13); **e** Epidermal cell area 2013 (CA-13); **f** Stomatal index 2013 (SI-13)

Data were transformed for normality as appropriate; Fig. 2 shows the frequency distributions of estimated biomass yield 2013, epidermal cell number per leaf and saccharification following transformation. Additional file 3: Table S1 shows the highly significant (*p* < 0.001) effect of genotype for all traits studied. Estimated biomass yield (Fig. 3a) varied between 0.05 and 6.52 tonnes ha^−1^ y^−1^. Similarly epidermal leaf cell number (Fig. 3b) varied between approximately 0.8 and 27 million cells per leaf. Glucose release (saccharification potential—Fig. 3c) as a percentage of PPT CWR (post pre-treatment, oven-dry, cell wall residue) varied from 2.2  to 19.58 %.

Site of Origin (SO) was significant for all traits with the exception of saccharification potential for which the SO effect narrowly missed significance with the random factors block and run included in the GLM. The presence of a significant block effect necessitated the use of block adjusted marginal means in downstream analyses. This wide genetic variation displayed in traits, related to both genotype and SO, highlights the potential of this natural germplasm collection to provide diversity for future selection and breeding efforts for this native European tree species. Figure 3 shows box plots for estimated biomass 2013 (EB-13), epidermal cell number per leaf area (CNPL-13), saccharification potential (SP-12), leaf area (LA-13), epidermal cell area (CA-13) and stomatal index (SI-13) by SO to give an indication of the extent and nature of the population-wide variation (boxplots for all other traits are available in supplementary Additional file 1: Figure S3).

**Fig. 4:**
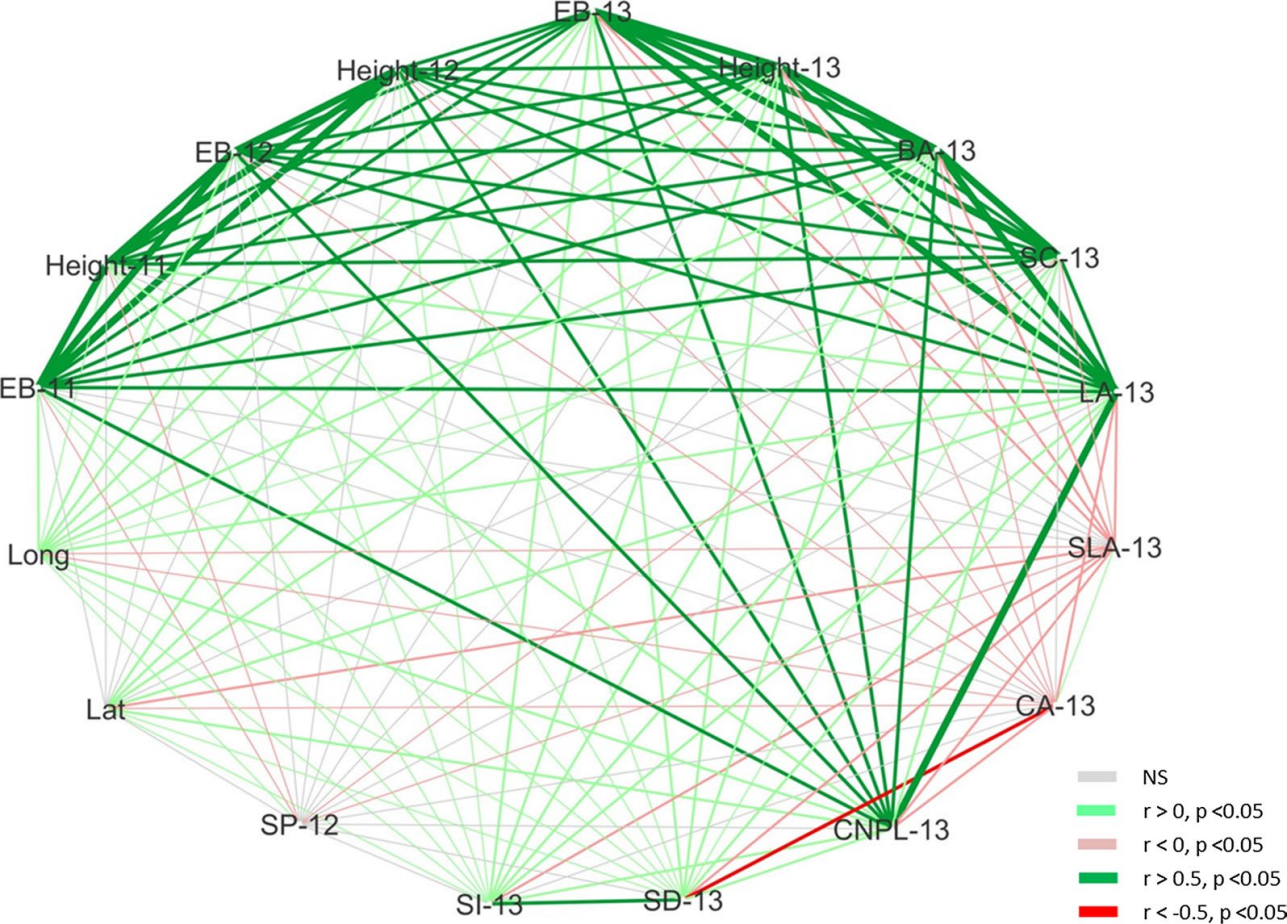
Pairwise trait correlations are visualised with line colours and widths conferred according to the strength and direction of Pearson’s correlation coefficient (*r*) between trait pairs. Non-significant correlations are depicted with *grey*, *point 1 lines*. Significant positive and negative correlations (*p* < 0.05) are depicted with *point 2 lines* coloured *light green* or *light red*, respectively. Strong positive and negative correlations (*r* > 0.5) are depicted with *point 3 lines* coloured *dark green* or *dark red*, respectively. Very strong positive correlations (*r* > 0.8) are also shown in *dark green* with *point 4 lines*

Figure 4 visualises the direction, magnitude and significance of Pearson’s r pairwise correlation between all traits and with latitude and longitude of genotype origin. Additional file 1: Figure S4A shows the correlation matrix itself with exact Pearson’s r and p-values displayed and trait heritabilities (*h*^2^) shown across the matrix diagonal. Additional file 1: Figure S4B depicts the same data as a scatter plot matrix. Biomass traits (estimated biomass yield, main stem height, basal area and primary stem count) show strong positive (*r* > 0.5) correlations within and where applicable between years as well as consistently significant, weak to moderate positive (*p* < 0.05, 0 < *r* < 0.5) correlations with longitude of origin. Biomass yield, height and basal area from 2013 also show a significant correlation with latitude. Leaf area (mature leaf size) 2013 shows a strong positive relationship with biomass yield from all years with the strongest correlation with EB-13 (*r* = 0.814, *p* < 0.001). It is also significantly positively correlated with both latitude (*r* = 0.422, *p* < 0.001) and longitude (*r* = 0.381, *p* < 0.001) of origin. By contrast specific leaf area (SLA-13) shows a moderate but significant negative correlation with EB-13 (r = −0.271, *p* < 0.001) and with leaf area (*r* = −0.283, *p* < 0.001). Epidermal cell number per leaf (CNPL-13) is naturally very tightly correlated with leaf area (*r* = 0.973, *p* < 0.001) and in turn shows a strong positive correlation with EB-11, 12 and 13. Cell area (CA-13) shows a weak negative correlation with EB-13 (*r* = −0.154, *p* < 0.001) and LA-13 (*r* = −0.227, *p* < 0.001) and a strong negative correlation with stomatal density (0.579, *p* < 0.001). Stomatal density and index (SD-13 and SI-13) show weak to moderate positive correlations with biomass traits in all years, leaf area and latitude and longitude of origin. Saccharification potential (SP-12) appears largely unrelated to the other traits measured with only very weak and in most cases non-significant correlations found. It shows no significant correlation with latitude and only a very weak relationship with longitude (*r* = 0.092, *p* = 0.021); as might be expected in view of its lacking an effect for SO in the GLM analysis.

**Fig. 5:**
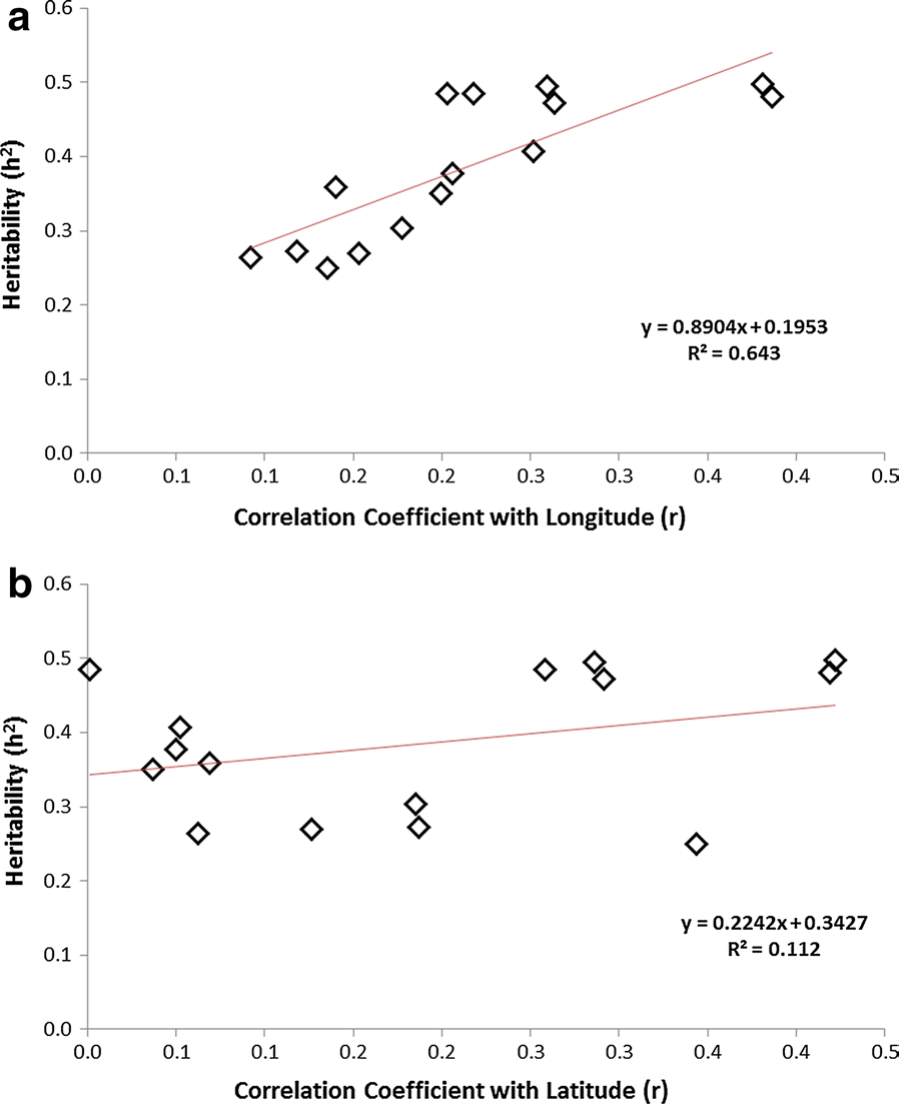
Trait heritabilities show significant positive regression with their correlation coefficients (*r*) for **a** longitude of origin (*r* ^2^ = 0.643) but not **b** latitude of origin (*r* ^2^ = 0.112)

Narrow-sense trait heritabilities (*h*^2^) ranged from 0.250 for SLA-13 to 0.497 for LA-13 (Additional file 1: Figure S4A). Heritability for biomass yield was moderate but consistent; ranging from 0.407 for EB-12 to 0.494 for EB-13. Figure 5 shows trait heritabilities did not regress significantly with their correlation coefficients (*r*) for latitude of origin (F_1, 13_ = 1.61, *p* = 0.226, *r*^2^ = 0.112) but regressed strongly for longitude (F_1, 13_ = 23.37, *p* < 0.001, *r*^2^ = 0.643).

### Population genetic structure

**Fig. 6:**
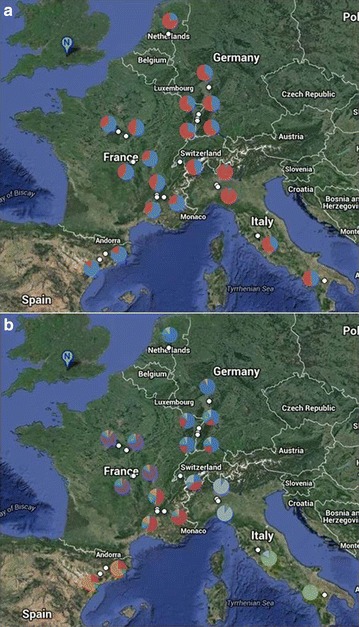
Satellite map of *P. nigra* association population subpopulation locations and their mean proportional cluster allocations from STRUCTURE for **a** *K* = 2 and **b** *K* = 7

STRUCTURE analysis after the method of Evanno et al. [83]. found the optimal value of *K* to be 2; i.e. the population of 714 genotypes is broken into 2 broad clusters shown in Fig. 6a. This model suggests the strongest differentiation in the population to be between the Spanish (Ebro) and Northern Italian (Ticino) subpopulations. However, for comparison, Fig. 6b shows the cluster memberships for *K* = 7 as previously proposed by Faivre-Rampant et al. [39]. While in contrast to the optimal model according to STRUCTURE, this visualisation serves to illustrate finer scale differentiation between subpopulations and the extent and nature of admixing which are less apparent from the *K* = 2 model. Thus, both models have something to offer in the interpretation of structure for this complex population. The Southern Italian (Basento) and Northern Italian (Ticino) genotypes are shown to belong to clearly distinct clusters with a degree of admixing in central Italy (Paglia). The German subpopulation (Kuhkopf) is strongly assigned to a unique cluster and is closely related to the more northerly Netherlands (NL) genotypes. Genotypes drawn from subpopulations on the France-Germany border (Rhinau, Strasbourg, Taubergiessen and Erstein) are also strongly assigned to this cluster but show some admixing with the Ticino subpopulation and with subpopulations in Southern and Central France as do the individuals from Dranse on the France-Switzerland border. The Central French subpopulations (Loire, Val Allier, Bonny and Guilly) are all predominantly assigned to their own cluster, whereas the Southern French (Drome and Ramieres) show more admixing; including with the distinctive Spanish populations (Ebro).

The PCA of the neutral genetic variance (2390 SNPs) revealed 7 significant PCs according to a broken stick model (see scree plot in Additional file 1: Figure S5); cumulatively explaining 12.2 % of the variation. These significant PCs were used in the GWAS P-model (model II). PCs 1, 2 and 3 explained 3.91, 2.18 and 1.95 %, respectively; a scatter plot of which (Additional file 1: Figure S6) shows good agreement with STRUCTURE (Fig. 6) with distinctive clusters for the Northern and Central/Southern Italian genotypes; a close relationship between German and Dutch individuals and the Spanish populations separated from the other nations by the more diffusely arrayed French. PCs 1–3 regress significantly with latitude and longitude of origin: PC1 with latitude (F_1, 710_ = 41.5, *p* < 0.001, *r*^2^ = 0.055); PC1 with longitude (F_1, 710_ = 319.28, *p* < 0.001, *r*^2^ = 0.310); PC2 with latitude (F_1, 710_ = 6.23, *p* = 0.013, *r*^2^ = 0.009); PC2 with longitude (F_1, 710_ = 257.92, *p* < 0.001, *r*^2^ = 0.267); PC3 with latitude (F_1, 710_ = 59.82, *p* < 0.001, *r*^2^ = 0.078); PC3 with longitude (F_1, 710_ = 9.35, *p* = 0.002, *r*^2^ = 0.013) (Additional file 1: Figure S7).

The PCA for all markers (7343 SNPs) also showed 7 significant PCs cumulatively explaining 16.3 % of the variation. PCs 1 and 2 explained 6.78 and 2.60 %, respectively, and the individual marker eigenvalues reveal clusters of top loading SNPs for both PCs. For PC1 3 of the top loaded SNPs are within a tight cluster (73 kb) on chromosome 10; 3 are within a 15 kb region of chromosome 6 while a further 8 of the top 15 (0.2 %) are located within a 1.5Mbp region of chromosome 17. For PC2 a group of 6 top loaded SNPs are located within a 15 kb region on chromosome 6 with a further 3 located in a 62 kb region of chromosome 8. Additional file 4: Table S2 shows the top 74 (1 %) loaded SNPs for PCs 1 and 2.

**Fig. 7:**
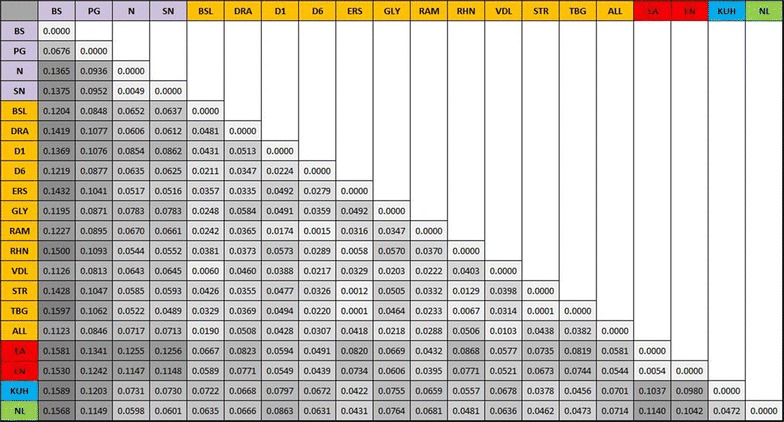
Genetic distance matrix (pairwise F_ST_) between 20 subpopulations of *P. nigra* association population. F_ST_ values are shaded according to magnitude (*white* to *dark grey*) with Italian subpopulations in *purple*; French in *orange*; Spanish in *red*; German in *blue* and Netherlands in *green*

Pairwise F_ST_ (calculated from the reduced, putatively neutral 2390 SNP marker set) between subpopulations (Fig. 7) shows that the Southern Italian (Basento) genotypes are the most genetically distant group with pairwise F_ST_ ranging from 0.112 (Val Allier) to 0.159 (Kuhkopf) against all other groups excepting the Central Italian (Paglia, F_ST_ = 0.068). As predicted from STRUCTURE the Spanish (Ebro) and Northern Italian (Ticino) are also more distantly related (FST range from 0.115 to 0.126). The German and Dutch subpopulations are again shown to be closely related (F_ST_ = 0.047). Within France F_ST_ is generally low with the greatest differentiation between Rhinau (France-Germany border) and the southerly Drome 1 (F_ST_ = 0.057).

The phenotypic PCA showed 2 significant PCs explaining 51.9 and 15.9 % of the phenotypic variance, respectively. The mean eigenvalues for these were calculated for the 20 sampled sites and the Euclidian distances calculated between them to produce a pairwise phenotypic distance matrix (Additional file 1: Figure S8) which shows the greatest distance between the Ticino subpopulations in Northern Italy and the Basento and Ebro subpopulations in the south of Italy and Spain, respectively. PC1 regressed weakly with longitude (*r*^2^ = 0.107) and trivially with latitude (*r*^2^ = 0.031) (Additional file 1: Figure S9).

The results of full and partial Mantel tests on the genetic (F_ST_), phenotypic and geographical distance matrices are shown in Table 2. There is strong positive correlation between genetic and geographic distance controlling for phenotypic distance (*r* = 0.844, *p* < 0.001) suggesting isolation by distance (IBD) and moderate positive correlation between genetic and phenotypic distance controlling for geographic distance (*r* = 0.304, *p* = 0.001) suggesting isolation by adaption (IBA).

**Table 2:** Mantel tests reveal IBD and IBA in European *P. nigra*

Mantel Test	Hypothesis	Corr. coefficient (*r*)	*p* value
(Gen, Geog)	–	0.855	<0.001
(Gen, Pheno)	–	0.385	<0.001
(Gen, Geog|Pheno)	IBD	0.844	<0.001
(Gen, Pheno|Geog)	IBA	0.304	0.001

Reports correlation coefficient (*r*) and *p* value (1000 permutations) for full Mantel tests investigating relationship between genetic and geographic (Gen, Geog) and genetic and phenotypic (Gen, Pheno) distance matrices as well as partial Mantel tests for isolation by distance (Gen, Geog|Pheno) and isolation by adaptation (Gen, Pheno|Geog)

### GWAS models and gene candidates

GWAS was conducted in TASSEL for all traits comparing 6 models: (1) a simple GLM with no population structure correction; (2) GLM with seven significant PCs from PCA of neutral genetic variance included as covariate (P-model); (3) GLM with Q-matrix (*K* = 2) from STRUCTURE (Q-model); (4) MLM with EMMA kinship matrix (K-model); (5) MLM with Q (*K* = 2) and kinship matrices (Q + K-model); (6) MLM with 7 PCs and kinship matrix (P + K model). The most appropriate model for each trait was then selected using BIC to compare log-likelihood values [95] between models on a trait-specific basis. The threshold for genome-wide significance was α = 8.79 × 10^−6^. Table 3 displays the numbers of significant SNPs for each trait under each model with the BIC-selected optimal model indicated (Additional file 5: Table S3). Manhattan and QQ plots for all traits for all models are provided in supplementary Additional file 1: Figure S10.

**Table 3:** Number of significant trait-SNP associations under all models

Trait	Model I	Model II	Model III	Model IV	Model V	Model VI
EB-11	925	0	27	0^a^	0	0
Height-11	600	1	6	1	1^a^	0
EB-12	1492	0	56	0	0^a^	0
Height-12	1385	0	26	0	0^a^	0
EB-13	1750	0	17	0	0^a^	0
Height-13	1517	1	8	1	1^a^	1
BA-13	1690	0	21	0	0^a^	0
SC-13	334	0	8	0^a^	0	0
LA-13	2803	1	162	2	0^a^	0
SLA-13	157	0^a^	42	0	0	0
CA-13	321	0	0^a^	0	0	0
CNPL-13	2908	2	146	3	1^a^	0
SD-13	705	0^a^	10	0	0	0
SI-13	99	0^a^	18	0	0	0
SP-12	1	0	1	0^a^	0	0

Number of significant trait-SNP associations at α < 8.79 × 10^−6^ under 6 possible models: (1) simple GLM (no genetic structure correction); (2) GLM with seven significant principal components of neutral genetic variation; (3) GLM with Q-matrix (*K* = 2) from STRUCTURE; (4) MLM with EMMA kinship matrix; (5) MLM with EMMA kinship and Q-matrix; (6) MLM with EMMA kinship and significant principal components of genetic variation. ^a^Indicates the optimal model selected by comparison of log-likelihoods using BIC

**Fig. 8:**
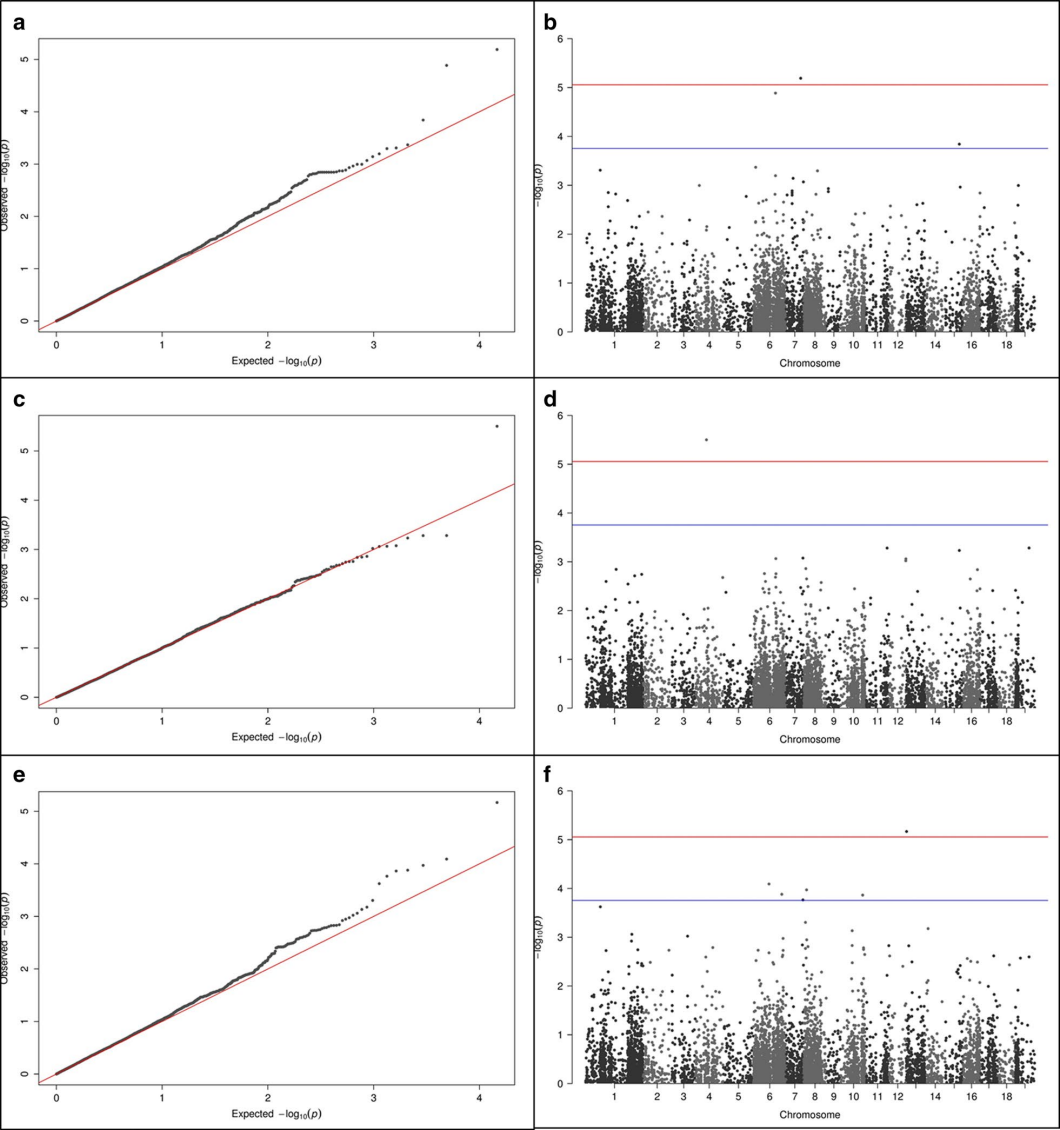
QQ and Manhattan plots for the Q + K (optimal) models for the 3 traits with SNPs reaching genome-wide significance.* Red* and* blue* lines on Manhattan plots illustrate genome wide (α < 8.79 × 10^−6^) and putative (α < 1.76 × 10^−4^) significance levels, respectively. **a** QQ plot for Height-11 associated SNP on chromosome 7; **b** Manhattan plot for Height-11 association; **c** QQ plot for Height-13 associated SNP on chromosome 4; **d** Manhattan plot for Height-13 associated SNP; **e** QQ plot for CNPL-13 associated SNP on chromosome 13; **f** Manhattan plot for CNPL-13 associated SNP

In no case was the simple model selected and in many cases this model saw a large number of false positives arising from the lack of population structure correction (see QQ plots in Additional file 1: Figure S10). Under the simple model the number of ‘significant’ associations ranged from 1 (SP-12) to 2908 (CNPL-13) with a mean of 1112. The number of such associations showed strong positive regression with trait heritability; F_1, 13_ = 31.14, *p* < 0.001, *r*^2^ = 0.706 (Additional file 1: Figure S11). The P-model was selected for 3 traits; the Q-model for 1 trait; the K-model for 3 traits and the Q + K model for the remaining 8. The P + K model was not selected for any traits and appeared to represent overfitting. Under these optimal models only 3 trait-marker associations reach genome-wide significance; 1 for Height-11, 1 for Height-13 and 1 for CNPL-13 (all Q + K model). Figure 8 displays Manhattan and QQ plots for these genome-wide significant associations. Table 4 shows the numbers of trait-marker associations for the optimal models at a range of significance thresholds.

**Table 4:** Significant trait-SNP associations under optimal model at three significance levels

Trait	Model	5 % (α < 8.79 × 10^−6^)	10 % (α < 1.76 × 10^−5^)	Putative (α < 1.76 × 10^−4^)
EB-11	K (IV)	0	0	1
Height-11	Q + K (V)	1	2	3
EB-12	Q + K (V)	0	0	2
Height-12	Q + K (V)	0	0	1
EB-13	Q + K (V)	0	0	0
Height-13	Q + K (V)	1	1	1
BA-13	Q + K (V)	0	0	0
SC-13	K (IV)	0	0	2
LA-13	Q + K (V)	0	0	2
SLA-13	P (II)	0	0	3
CA-13	Q (III)	0	0	5
CNPL-13	Q + K (V)	1	1	6
SD-13	P (II)	0	0	1
SI-13	P (II)	0	0	1
SP-12	K (IV)	0	0	1

Number of significant trait-SNP associations under the optimal model for each trait at 3 significance levels: 5 % α = 0.05/5690 (8.79 × 10^−6^); 10 %) α = 0.1/5690 (1.76 × 10^−5^); Putative) α = 1/5690 (1.76 × 10^−4^)

**Fig. 9:**
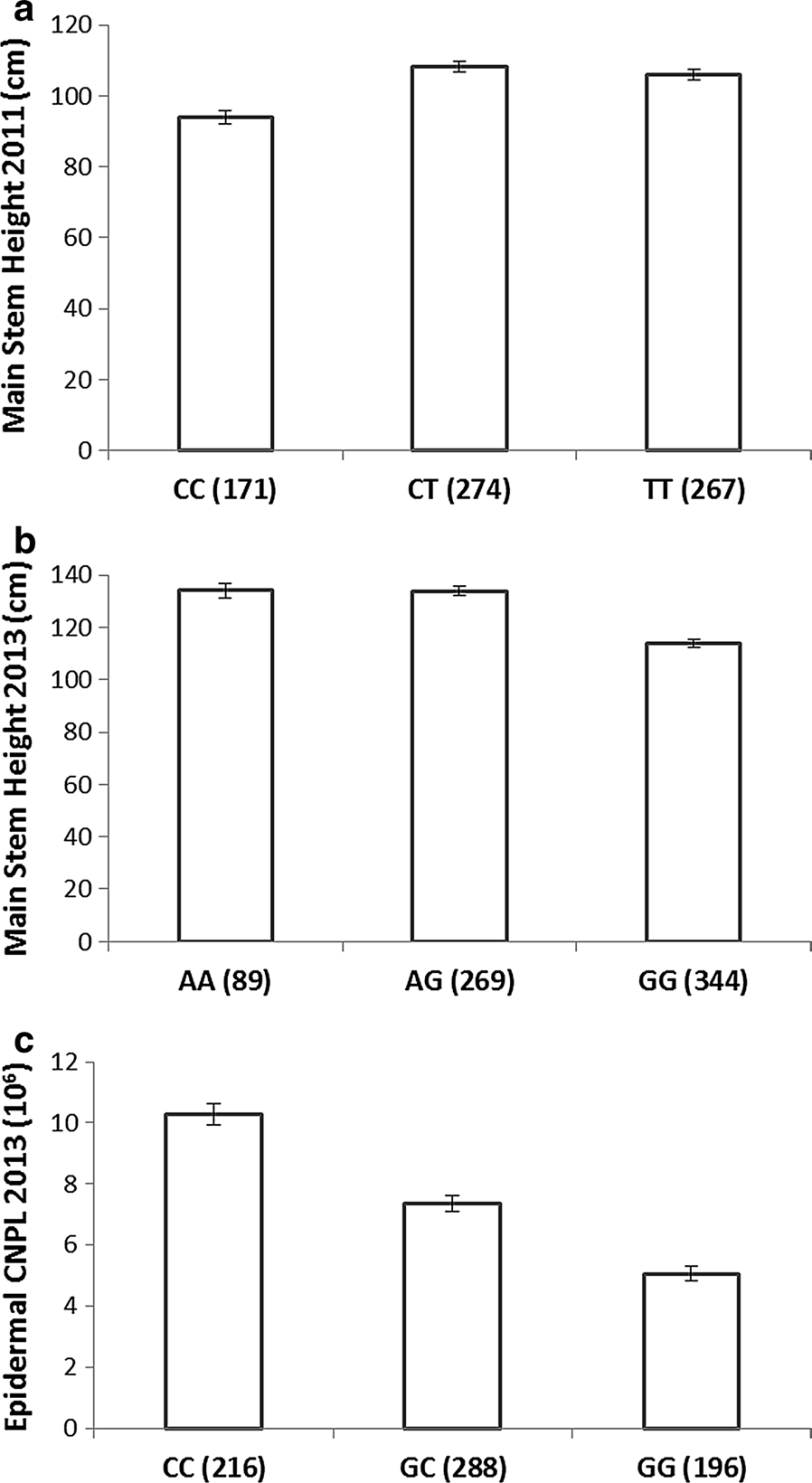
Bar plots of raw effects sizes (with standard *error bars*) for each trait-associated SNP with genome-wide significance from trait-specific optimal model for **a** Height-11 associated SNP; **b** Height-13 associated SNP and **c** CNPL-13 associated SNP. The *x-axis* of each plot gives the identity of each allelic variant (MM, MN or NN) with its sample size (*n*) within the population given in adjacent *brackets*

The 3 associated SNPs for Height-11 (also putatively associated with Height-12), Height-13 and CNPL-13 (also putatively associated with LA-13) are located on chromosome 7 within an intron of the gene POPTR_0007s11900; chromosome 4 within the first exon of the gene POPTR_0004s10800 (synonymous) and 1 kb to the 3′ end of the gene POPTR_0013s00340, respectively. The bar plots in Fig. 9 display the relationship between each marker and its associated trait. All 29 significantly and putatively trait-associated SNPs and their gene candidates are shown in Table 5.

**Table 5:** Trait-marker associations and candidate genes reaching genome-wide or putative significance under optimal models

Trait	SNP	Chromosome	Position (bp)	*p* value	Candidate gene	Additional information
EB-11	SNP_IGA_6_17929363	6	17,822,770	9.55E−05	POPTR_0006s18990	CNGC17; ATCNGC17; calmodulin binding/cyclic nucleotide binding/ion channel
Height-11	*SNP_IGA_7_12319871*	*7*	*12,250,804*	*6.46E−06*	*POPTR_0007s11900*	*Unknown protein* ^*a*^
Height-11	SNP_IGA_6_18338146	6	18,228,999	1.30E−05	POPTR_0006s19240	GAE1; UDP-glucuronate 4-epimerase/catalytic+
Height-11	SNP_IGA_15_11900175	15	11,834,554	1.44E−04	POPTR_0015s11190	Unknown protein
EB-12	SNP_IGA_6_8443540	6	8,388,882	9.47E−05	POPTR_0006s11060	ATH9 (thioredoxin H-type 9)
EB-12	SNP_IGA_7_993475	7	987,055	1.54E−04	POPTR_0007s01700	GLX2-4 (glyoxalase 2-4); hydrolase/hydroxyacylglutathione hydrolase/zinc ion binding
Height-12	SNP_IGA_7_12319871	7	12,250,804	4.70E−05	POPTR_0007s11900	Unknown protein#
Height-13	*PnCOL2_703*	*4*	*9,357,150*	*3.16E−06*	*POPTR_0004s10800*	*COL2 (constans*-*like 2); transcription factor/zinc ion binding* ^a^
SC-13	SNP_IGA_1_44937224	1	44,670,745	1.05E−04	POPTR_0001s44200	ATK3 (ARABIDOPSIS THALIANA KINESIN 3); ATPase/microtubule binding/microtubule motor
SC-13	SNP_IGA_6_7875360	6	7,824,407	1.55E−04	POPTR_0006s10480	FER1; ATFER1; ferric iron binding/iron ion binding
LA-13	SNP_IGA_13_111400	13	110,728	7.83E−05	POPTR_0013s00340	RCI2A (RARE-COLD-INDUCIBLE 2A)#
LA-13	SNP_IGA_8_2418643	8	2,403,351	8.24E−05	POPTR_0008s04290	Unknown protein
SLA-13	SNP_IGA_14_3311885	14	3,293,256	8.14E−05	POPTR_0014s04150/POPTR_0014s04160	Unknown protein/PEX11A (PEROXIN 11A)
SLA-13	SNP_IGA_19_2255781	19	2,244,885	1.53E−04	POPTR_0019s02450	SWIM zinc finger protein-related
SLA-13	SNP_IGA_6_23541394	6	23,399,832	1.73E−04	POPTR_0006s24880	PP2C; protein phosphatase 2C family protein
CA-13	SNP_IGA_6_3818713	6	3,794,879	7.31E−05	POPTR_0006s05370	Unknown protein
CA-13	PnCOL2_69	4	9,356,516	1.14E−04	POPTR_0004s10800	COL2 (constans-like 2); transcription factor/zinc ion binding#
CA-13	SNP_IGA_1_29550802	1	29,371,580	1.20E−04	POPTR_0001s30950	RD21 (responsive to dehydration 21); cysteine-type endopeptidase/cysteine-type peptidase
CA-13	LG_X_35_SNP_325	10	14,394,839	1.51E−04	POPTR_0010s14950	BAS1 (PHYB ACTIVATION TAGGED SUPPRESSOR 1); oxygen binding/steroid hydroxylase
CA-13	LG_X_35_SNP_490	10	14,395,004	1.51E−04	POPTR_0010s14950	As above
CNPL-13	*SNP_IGA_13_111400*	*13*	*110,728*	*6.81E−06*	*POPTR_0013s00340*	*RCI2A (RARE*-*COLD*-*INDUCIBLE 2A)* ^*a*^
CNPL-13	SNP_IGA_6_12938719	6	12,856,743	8.11E−05	POPTR_0006s15470	Bacterial transferase hexapeptide repeat-containing protein
CNPL-13	SNP_IGA_8_2308644	8	2,294,048	1.07E−04	POPTR_0008s04110	AGL62 (Agamous-like 62); DNA binding/transcription factor
CNPL-13	SNP_IGA_6_23601531	6	23,459,494	1.32E−04	POPTR_0006s24980	Unknown protein
CNPL-13	SNP_IGA_10_18449865	10	18,340,999	1.37E−04	POPTR_0010s20920	Immunophilin, putative/FKBP-type peptidyl-prolyl cis–trans isomerase, putative
CNPL-13	SNP_IGA_7_14212007	7	14,130,925	1.72E−04	POPTR_0007s14310	AGL22 (Agamous-like 22); SVP; transcription factor/translation repressor, nucleic acid binding
SD-13	SNP_IGA_6_11135816	6	11,064,511	1.67E−04	POPTR_0006s13890	TES (TETRASPORE); microtubule motor
SI-13	SNP_IGA_6_8990445	6	8,932,413	1.24E−04	POPTR_0006s11720	DML1 (DEMETER-LIKE 1); DNA N-glycosylase/DNA-(apurinic or apyrimidinic site) lyase/protein binding
SP-12	SNP_IGA_1_31674244	1	31,482,266	1.60E−04	POPTR_0001s33290	Zinc finger (DHHC type) family protein

Under optimal models there are 29 SNPs (representing 25 candidate genes) reaching at least the putative significance level (α < 1.76 × 10^−4^) of which 4 are significant at *p* < 0.1 (indicated by a +) and three are significant at *p* < 0.05 (in italic typeface and indicated by ^a^). Genes putatively associated with one trait whilst significantly associated with another at *p* < 0.05 are indicated by a #

## Discussion

The bioenergy trait data reported here demonstrates the extent of phenotypic variation within this *P. nigra* association mapping population with greater than tenfold differences between genotypic extremes for many key traits (e.g. biomass yield, leaf area and saccharification potential, Fig. 3) and a significant genotypic effect for all traits measured. This provides important novel data for a *Populus* that is native to Europe and a source of previously uncharacterised variation that may be harnessed in future for selection and breeding pipelines. Importantly, biomass yield traits were consistent across time with strong correlations within and between growing seasons and across a coppice cycle and possess moderate narrow sense heritabilities (Additional file 1: Figure S4). This is an important finding since it suggests that simple to measure traits such as leaf size and leaf cell number may be considered as early diagnostic indicators of tree yield in a long-lived crop that may take several years to reach maturity. In addition, these are also promising qualities for association genetics within the population, enabling us to identify informative candidate genes for future molecular breeding efforts [99] for improved biomass yield in *Populus*. Irrespective of end use, consistent high biomass productivity is a key trait and this population is a useful resource to elucidate the genetics of biomass and biomass-related traits. For liquid fuel applications wood quality and biomass digestibility are also important considerations and a research priority [100, 101]. The limited correlations between saccharification potential and biomass traits shown in Fig. 4 (strongest relationship is with biomass yield 2011, *r* = −0.107) are encouraging since they imply that gains in biomass yield may be obtainable without negative impacts on the quality traits underpinning feedstock processing.

The strong correlation between leaf area (individual leaf size) and biomass yield in 2013 (*r* = 0.814) has been previously reported in *Populus* [61, 102] in pedigree mapping populations and here we confirm the value of leaf area as a highly heritable (*h*^2^ = 0.497) diagnostic indicator of biomass productivity [103]. Interestingly, epidermal cell number was significantly more heritable than epidermal cell area (*h*^2^ = 0.480 and 0.270, respectively) and showed a far stronger correlation with total leaf area (Pearson’s *r* = 0.973 and −0.227, respectively). Previous research [65] in this population has also shown that leaf cell production rather than cell expansion is highly heritable and the role of cell production in the development of large leaves is well established [104]. This is likely due to cell expansion being driven by biophysical events in the cell whilst cell production is driven by the cell cycle and signalling which are strongly genetically determined and hence highly heritable [105]. The cell division phase of leaf development, which follows the emergence of the primordium from the shoot apical meristem (SAM), is central to determining the total number of cells in the leaf and hence it’s final, developed size. The extent of cell production in this phase is dependent on the rate of passage through the cell cycle which is controlled by proteins involved in DNA replication and mitosis and those that regulate them; e.g. cyclins, ubiquitin ligases and gibberellin oxidases [105]. The transgenically altered expression of proteins involved in cell cycle regulation has been shown to impact final leaf size in Arabidopsis [106, 107] and if such genes can be identified in bioenergy *Populus* they may prove valuable candidates for leaf development and biomass yield.

Figure 3 shows the Spanish and Southern Italian subpopulations had the lowest biomass yields and smallest leaves with subpopulations from northern Italy, Germany, The Netherlands and the French–German border showing the highest biomass production and largest leaves. It is possible that genotypes originating from regions geographically closer and climatically similar to Northington are performing optimally in this experiment. Such G × E interactions can only be investigated through multiple site or environment trials, however, which can be challenging in large populations such as the one described here although current research is underway to test this population at two levels of soil moisture. Furthermore, there is clear evidence in this case that phenotype is strongly influenced by geographical factors with all traits with the exception of saccharification potential (*p* = 0.056) showing a strongly significant (*p* < 0.001) effect for Site of Origin (SO). Additionally, all traits show a weak to moderate correlation with longitude of origin and 9 (of 15) show a significant relationship with latitude of origin. Further evidence that phenotypic variation is more closely aligned with longitude than latitude (i.e. trait variation follows a predominantly east–west cline) is provided in Fig. 5 displaying the far greater strength of the regression of trait heritabilities against their correlation coefficients with longitude (*r*^2^ = 0.643) than latitude (*r*^2^ = 0.112). The first principal component of the PCA of phenotypic variance also showed a stronger regression with longitude (*r*^2^ = 0.104) than latitude (*r*^2^ = 0.032) (Additional file 1: Figure S9). This assessment is supported by the PCA of the neutral genetic variance; the first 2 principal components thereof showing only a trivial relationship with latitude (*r*^2^ = 0.055 and 0.009, respectively) but a clear relationship with longitude (*r*^2^ = 0.310 and 0.267 ,respectively) (Additional file 1: Figure S7). This result contrasts with a common garden study in a *P. trichocarpa* population, drawn from the west coasts of Canada and the USA with a latitudinal range of 44–59.6°N, which reported strong correlations between latitude and many biomass traits including height, branching and growth rate [49].

The PCA of the full marker set (i.e. including markers lacking complete information and potentially under selective pressure) identified clusters of markers with highly weighted eigenvalues for the first 2 PCs (Additional file 4: Table S2). These clusters on chromosomes 6, 10 and 17 for PC1 and 6 and 8 for PC2 may contain genes that have experienced strong selective pressure as genotypes adapted to their environment and as such could merit further investigation [49].

Table 2 presents evidence of strong IBD and moderate IBA in this population with partial Mantel tests showing significant positive correlations between genetic and geographical distance when controlling for phenotypic distance (IBD, *r* = 0.844, *p* < 0.001) and between genetic and phenotypic distance when controlling for geographical distance (IBA, *r* = 0.304, *p* = 0.001). A good example of IBD is provided by contrasting the Basento (S Italy) and Ebro (Spain) subpopulations which show high pairwise F_ST_ (0.1530 and 0.1581, see Fig. 7) but only a small phenotypic distance (Additional file 1: Figure S8). These results confirm those using a much smaller set of microsatellite data [64] and support the proposition that this pattern of IBD may result from isolation by colonisation (IBC) as *P. nigra* recolonized central Europe from refugia following the last glacial maximum [108]. Cottrell et al. [108] utilised restriction fragments of chloroplast DNA from European *P. nigra* and found that France was most likely recolonized from the Iberian Peninsula (i.e. Spain) whilst Germany and the Lowlands (including The Netherlands) were likely recolonized from the Italian and Balkan Peninsulas. This is supported by both microsatellite data [64] and the far more extensive SNP data described here.

Figure 8 shows Manhattan and QQ plots for the 3 trait-marker associations reaching genome-wide significance in the optimal models (all Q + K in these instances). Visual inspection of the QQ plots shows these associations to be robust with population structure fully controlled. Figure 9 shows the raw effect size for each marker on its associated trait. POPTR_0007s11900 (significantly associated with Height-11 and putatively associated with Height-12) is a gene of unknown function, however, the UniProt database [109] suggests it to contain multiple transmembrane helices. POPTR_0004s10800 (significantly associated with Height-13 and putatively associated with epidermal cell area 13) is a COL2 (constans-like 2) transcription factor with twin zinc ion binding B-box domains. Its Arabidopsis ortholog AT5G15840 (BBX1) is one of 21 such twin B-box transcription factors in this model species [110] (an additional 11 having a single B-box). Members of this closely structurally related but functionally diverse family have been implicated in the control of flowering time [111] and growth [112]. Excitingly, one member (AT4G38960, BBX19) has been recently demonstrated to act as a positive regulator of hypocotyl extension in Arabidopsis [112] (mediated through its action as a negative regulator of photomorphogenesis) and thus it is feasible that POPTR_0004s10800 is making a contribution to growth in *Populus*. Encouragingly, in a recent glasshouse trial of 3 diverse genotypes drawn from this population, POPTR_0004s10800 was shown to be differentially expressed in developing xylem (Additional file 1: Figure S12); with significantly (*p* = 0.005) higher expression levels seen in the genotype possessing the “A” allele associated with greater height in this study (also see Fig. 9b). POPTR_0013s00340 (significantly associated with CNPL-13 and putatively associated with the closely correlated LA-13) is similar to hydrophobic protein RCI2A; its Arabidopsis ortholog AT3G05880 has been linked to the stress response and cold tolerance [113, 114]. Additional file 1: Figure S12 shows POPTR_0013s00340 to be differentially expressed in both developing xylem and leaf tissue in glasshouse grown *P. nigra*. These functional data provide another line of evidence to support these genes’ role in biomass determination in *Populus.* None of these genes has been previously linked to biomass yield or bioenergy in the literature and as such they represent novel candidates for further work.

Table 5 shows all 29 SNPs (25 genes) reaching genome-wide or putative significance for yield traits, leaf area, epidermal cell size, cell number per leaf and stomatal patterning. Five of the genes are of entirely unknown function and while none have been previously implicated in bioenergy traits in *Populus*; there are several genes of particular interest among the putative candidates which have been characterised in Arabidopsis. POPTR_0006s19240 is putatively associated with Height-11 (significant genome-wide association at *p* < 0.1). Its Arabidopsis ortholog AT4G30440 (known as GAE1) is a UDP-glucoronate 4-epimerase enzyme involved in pectin biosynthesis. When GAE1 expression was suppressed in conjunction with its homolog GAE6 in Arabidopsis the mutants displayed a mutant phenotype comprising slightly reduced size, leaf brittleness and suppressed immunity [115]. POPTR_0006s11060 is putatively associated with estimated biomass yield in 2012 and its Arabidopsis ortholog AT3G08710 is better known as ATH9. ATH9 is a membrane associated thioredoxin which has been shown to be plasma membrane associated and mobile between cells; suggesting a role in cell communication. A loss of function mutation in this gene in Arabidopsis resulted in impaired growth and development [116]. Two linked SNPs in POPTR_0010s14950 are putatively associated with epidermal cell area. This gene’s Arabidopsis ortholog is BAS1 (AT2G26710) which, like BBX19 discussed above, has been shown to play a role in the regulation of photomorphogenesis in Arabidopsis and thus impact upon hypocotyl elongation and cotyledon expansion [117]. POPTR_0008s04110 is putatively associated with epidermal cell number per leaf. Interestingly, its ortholog in Arabidopsis (the transcription factor Agamous-like 62, AGL62) has been demonstrated as essential in endosperm development where it acts as a regulator of cellularization in the plant embryo and is expressed strongly in the syncytial phase of mitotic cell production [118, 119]. In view of the strong relationship between biomass yield and leaf area (which appears to be driven largely by epidermal cell production) candidate genes for the control of mitotic cell division in the developing leaf could be very valuable as discussed above. While there is no report in the literature at present for such a role for POPTR_0008s04110; the poplar eFP browser [120] does show it to be strongly expressed in young leaves, an expression level that drops markedly in developed leaves. Another gene putatively associated with CNPL-13, POPTR_0007s14310, is also orthologous to an Agamous-like transcription factor (AGL22) in Arabidopsis. This gene is known as Short Vegetative Phase (SVP) due to its well established role as a repressor of floral development; acting to regulate cell differentiation and floral meristem determination [121]. Thus, we have identified a suite of candidate genes that may be explored further using reverse genetic approaches, such as those provided by CRISPR-CAS technology already available in *Populus* [122].

The tendency for uncorrected population structure to cause inflated and false positive test statistics for trait-marker associations is well documented and much effort has been invested in developing robust methodologies for its control [56, 123, 124]. Such structure has posed a challenge to researchers utilising the 34 K genotyping array developed for *P. trichocarpa* [44]. Publications for biomass yield; wood quality; ecophysiology and disease resistance traits in this species have variously employed kinship matrices; principal components of genetic variance and Q-matrices to ensure the reporting of robust associations [45–48]. Here, a large excess of false positives was observed when a simple, uncorrected model was employed with larger numbers of inflated values occurring for more highly heritable traits (e.g. LA-13, see Table 3). It was thus considered important to explore structure more thoroughly and its impact on trait associations was interrogated using a strategy similar to that of McKown et al. [47]. We used BIC to compare log-likelihoods between GWAS models using no correction; PCs, Q-matrix, kinship matrix or both Q-matrix/PCs and K-matrix together. It appears that this *P. nigra* association population is more highly structured than that for *P. trichocarpa*. McKown et al. [47] found that in all cases the simple, P or Q-models were sufficient and no traits required the more stringent K, Q + K or P + K models unlike in this work. They also found only PC1 of the neutral genetic variance to be significant opposed to the first 7 PCs in this instance. It follows that the numbers of significant associations discovered in the studies described in *P. trichocarpa* vastly exceed those reported here. Whilst this can be partly attributed to the superior numbers of SNPs on the 34 K chip and the greater numbers of traits phenotyped it is also likely that the lack of strong population structure in the *P. trichocarpa* association population is enormously beneficial in preventing over-correction by the application of more stringent models to control for stratification. Nevertheless, the associations provided above can be considered as robust for this out-breeding tree native to Europe and provide a firm basis for further proof of concept testing.

## Conclusions

Our research on native European black poplar provides a significant foundation for the development of commercial native trees for bioenergy and has identified important early diagnostic traits (leaf size and cell number) underpinning robust yield assessments over several years. We have been able to link these biomass traits to a set of candidate genes, varying from strong to putative but worthy of further investigation, that show differential expression in preliminary validation analysis. Although population structure; relatively low marker density and rapid decay of LD [39] have rendered association genetic analysis challenging; 3 robust associations were identified at full genome-wide significance for important biomass traits and 22 further genes are considered putative. It has been estimated [39] that 67–134K SNPs would be necessary to tag the entire genome (assuming an even marker density genome-wide) and, whilst greatly in excess of those available to this work, this number is within the scope of modern genotyping-by-sequencing (GBS) methodologies [42, 125]. A future GWAS in this population with a larger marker set more fully capturing the gene space may, therefore, be more fruitful in terms of the numbers of trait-marker associations obtained; notwithstanding increased penalisation for multiple testing corrections. Nevertheless, this study has provided valuable information regarding the likely challenges of working within this population and identified a modest number of gene candidates for bioenergy arising from the 12K array. Earlier work on the population’s genetic structure, based on small numbers of amplified DNA fragments and neutral markers prior to Next Generation Sequencing (NGS) approaches, has also been confirmed [54, 64, 108].

## Additional files

10.1186/s13068-016-0603-1 containing supplementary figures S1 to S11. Supplementary figure legends are contained within the file.
10.1186/s13068-016-0603-1 containing the SNP marker set derived from the 12 K Illumina array genotyping and employed here for GWAS.
10.1186/s13068-016-0603-1 containing supplementary table S1; showing the results of the general linear model run for all phenotypic traits.
10.1186/s13068-016-0603-1 containing supplementary table S2; depicting the top 1 % loading SNPs for the first 2 principal components of the genetic variation.
10.1186/s13068-016-0603-1 containing supplementary table S3; showing the BIC values for optimal model selection for GWAS.
10.1186/s13068-016-0603-1 containing supplementary Table 1096 S4; reporting the raw and normalised RNAseq count data used for supplementary Figure S12.

## Abbreviations

2G: 2nd generation
AFLP: amplified fragment length polymorphism
BIC: Bayesian Information Criterion
CA: cell area
CNPL: epidermal cell number per leaf
DNA: deoxyribonucleic acid
EB: estimated biomass
ECD: epidermal cell density
EMMA: efficient mixed model association
GLM: general linear model
GOD-POD: glucose oxidase peroxidase
GWAS: genome wide association study
G × E: genetic × environment
IBA: isolation by adaption
IBD: isolation by distance
LA: leaf area
LD: linkage disequilibrium
Meff: effective marker number
MLM: mixed linear model
PC: principal component
PCA: principal component analysis
PPT CWR: post pre-treatment cell wall residue
QQ: quantile quantile
QTL: quantitative trait loci
SAM: shoot apical meristem
SD: stomatal density
SI: stomatal index
SLA: specific leaf area
SNP: single nucleotide polymorphism
SO: site of origin
SP: saccharification potential
SRC: short rotation coppice
SRF: short rotation forestry
SVI: stem volume index
