# Supplementary material for: Biomass traits and candidate genes for bioenergy revealed through association genetics in coppiced European Populus nigra (L.)
Source: Biotechnol Biofuels. 2016 Sep 8;9(1):195. doi: 10.1186/s13068-016-0603-1 (PMC5017058; doi:10.1186/s13068-016-0603-1)
Supplement: Supplementary file 1 — 10.1186/s13068-016-0603-1 containing supplementary figures S1 to S11. Supplementary figure legends are contained within the file. [file 13068_2016_603_MOESM1_ESM.pptx]

## Slide 1
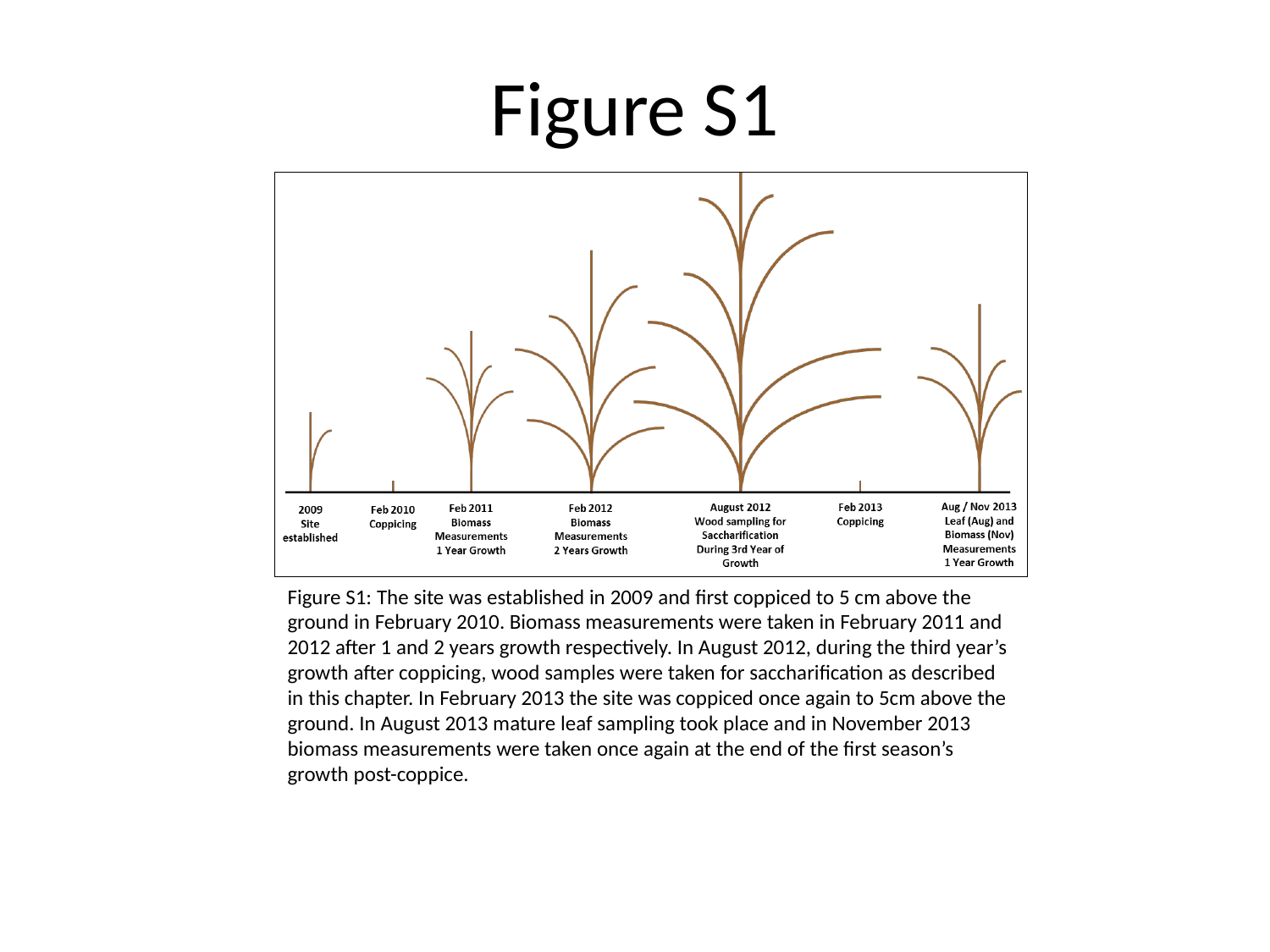

# Figure S1
Figure S1: The site was established in 2009 and first coppiced to 5 cm above the ground in February 2010. Biomass measurements were taken in February 2011 and 2012 after 1 and 2 years growth respectively. In August 2012, during the third year’s growth after coppicing, wood samples were taken for saccharification as described in this chapter. In February 2013 the site was coppiced once again to 5cm above the ground. In August 2013 mature leaf sampling took place and in November 2013 biomass measurements were taken once again at the end of the first season’s growth post-coppice.

## Slide 2
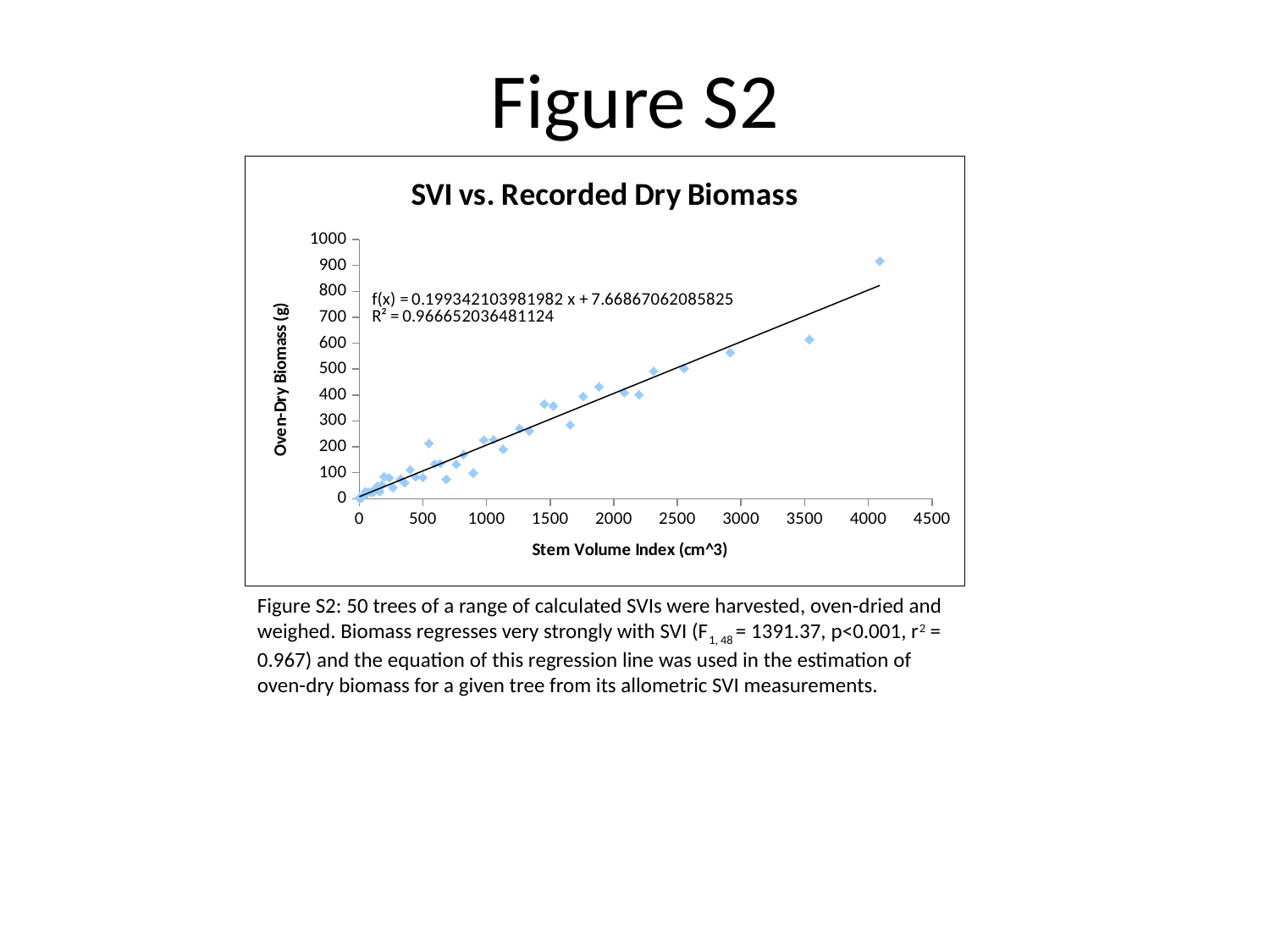

# Figure S2
### Chart: SVI vs. Recorded Dry Biomass
| Category | OD Biomass (g) |
|---|---|Figure S2: 50 trees of a range of calculated SVIs were harvested, oven-dried and weighed. Biomass regresses very strongly with SVI (F1, 48 = 1391.37, p<0.001, r2 = 0.967) and the equation of this regression line was used in the estimation of oven-dry biomass for a given tree from its allometric SVI measurements.

## Slide 3
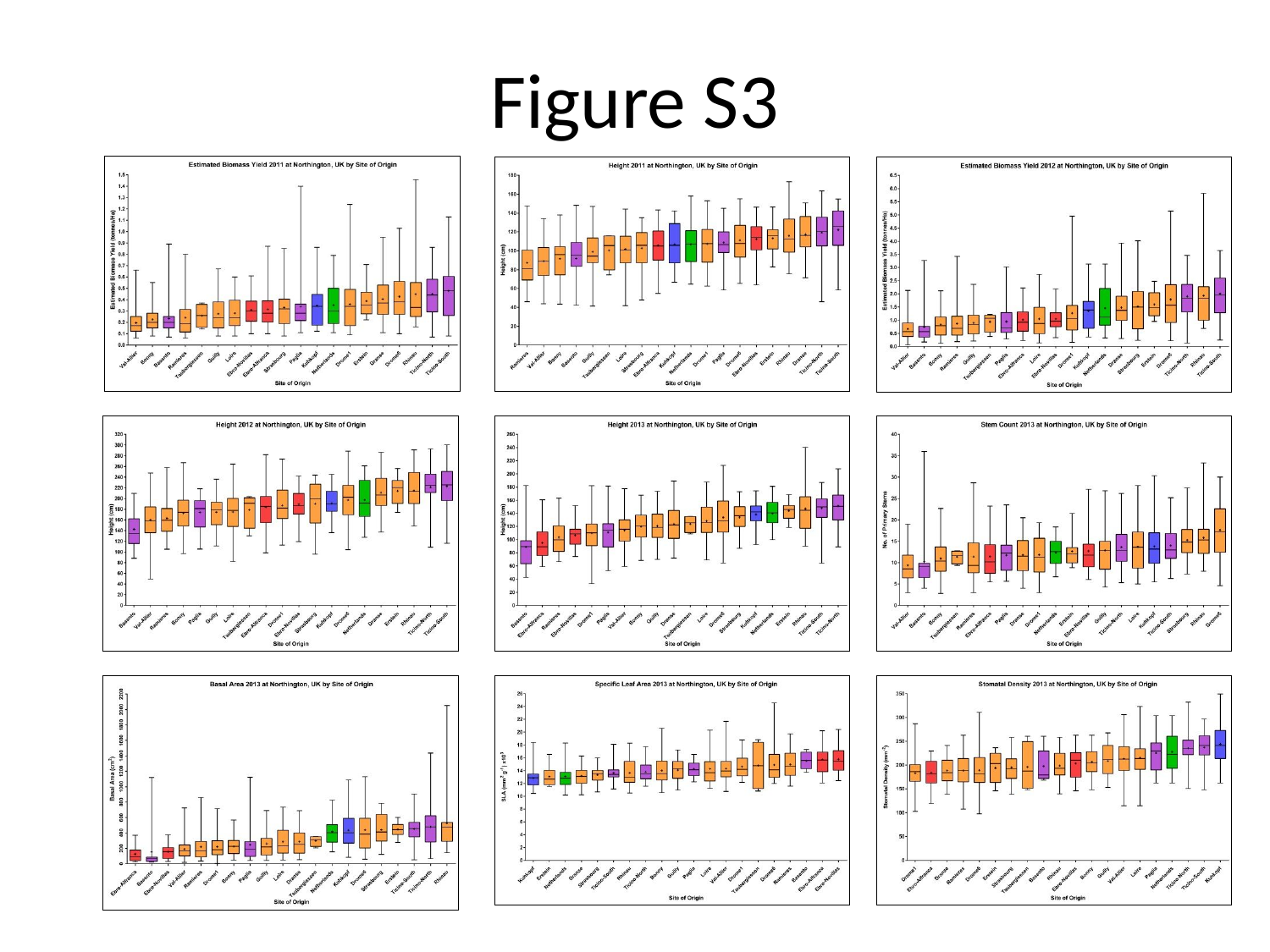

# Figure S3

## Slide 4
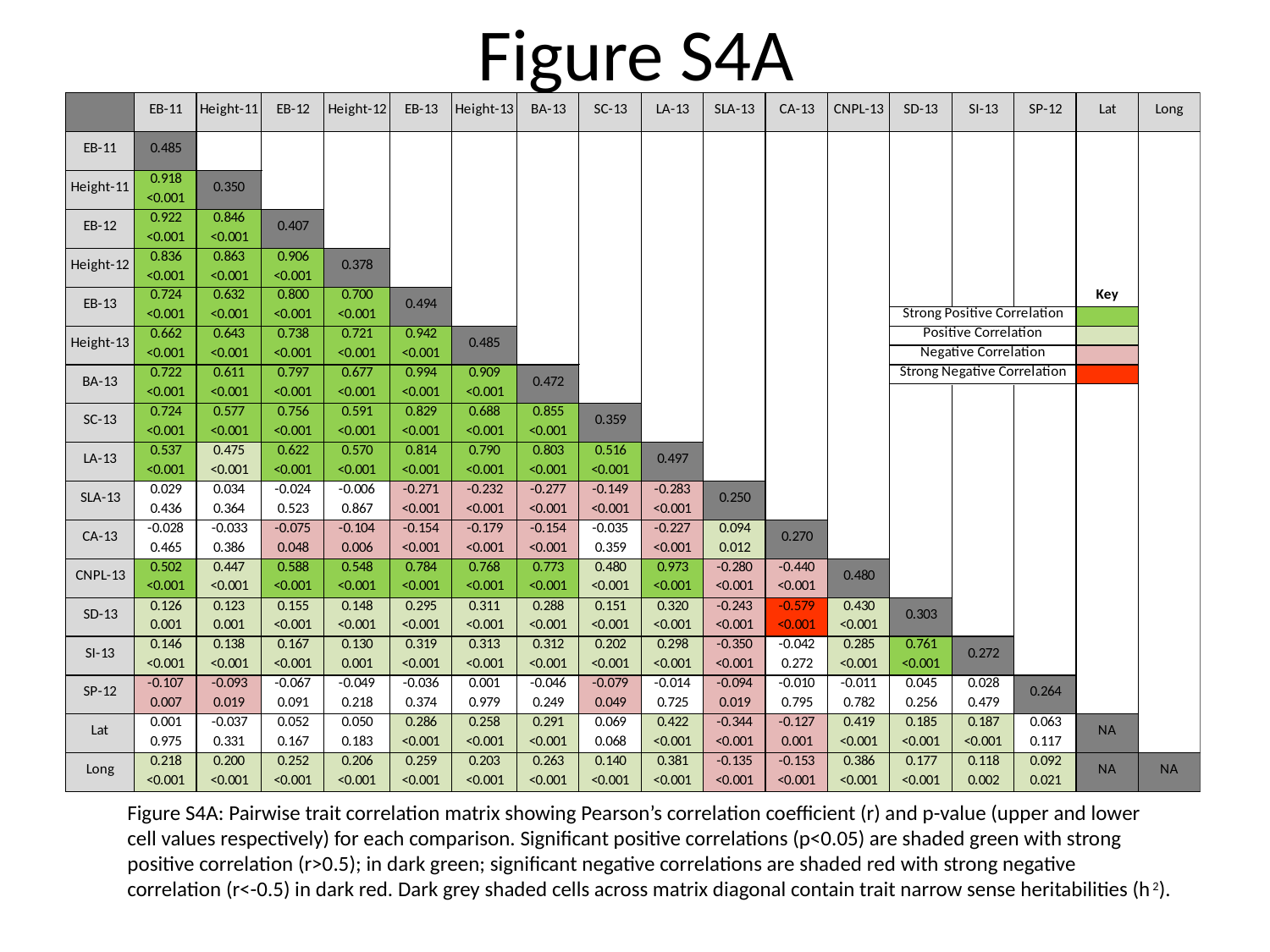

# Figure S4A
Figure S4A: Pairwise trait correlation matrix showing Pearson’s correlation coefficient (r) and p-value (upper and lower cell values respectively) for each comparison. Significant positive correlations (p<0.05) are shaded green with strong positive correlation (r>0.5); in dark green; significant negative correlations are shaded red with strong negative correlation (r<-0.5) in dark red. Dark grey shaded cells across matrix diagonal contain trait narrow sense heritabilities (h2).

## Slide 5
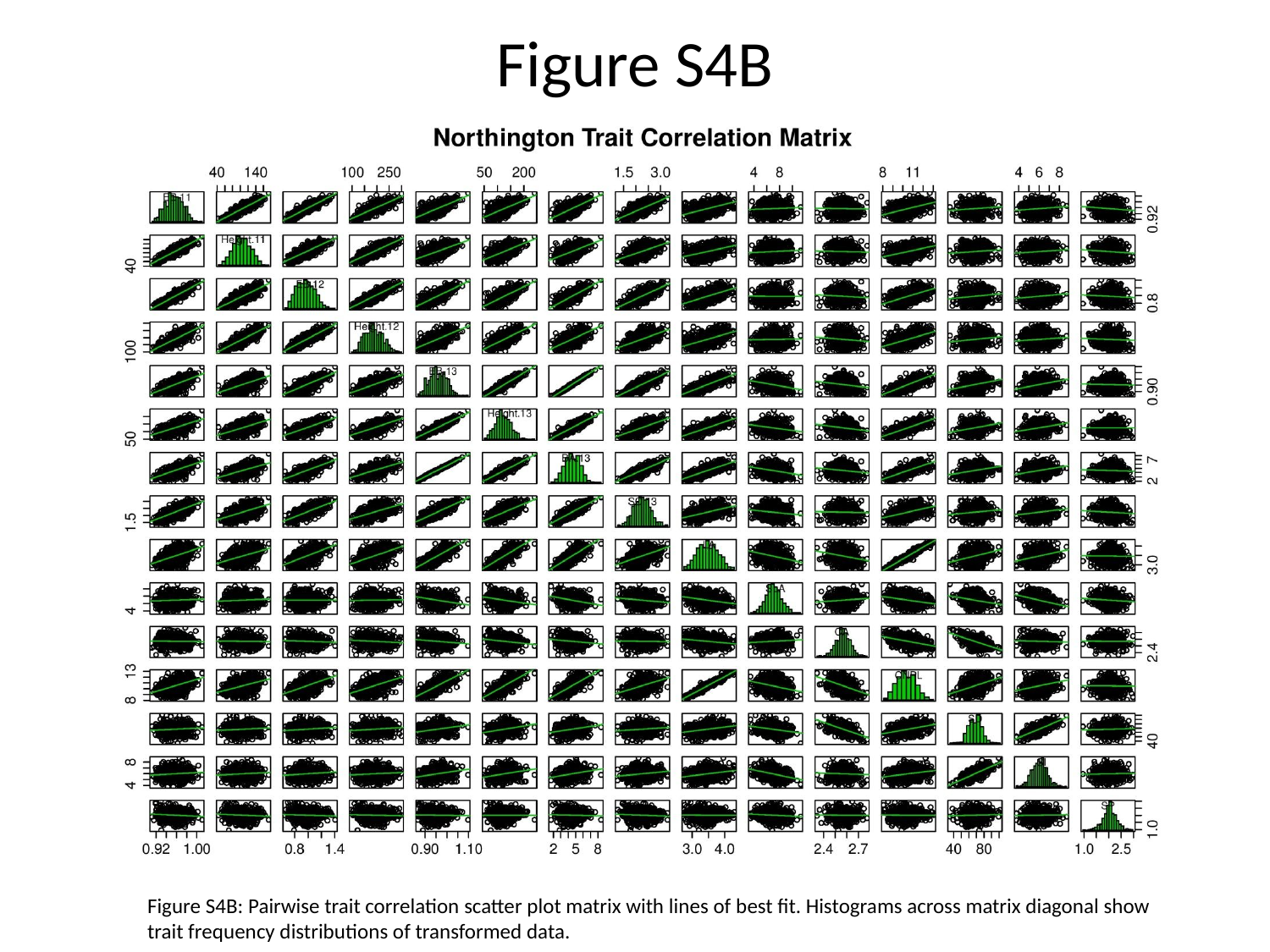

# Figure S4B
Figure S4B: Pairwise trait correlation scatter plot matrix with lines of best fit. Histograms across matrix diagonal show trait frequency distributions of transformed data.

## Slide 6
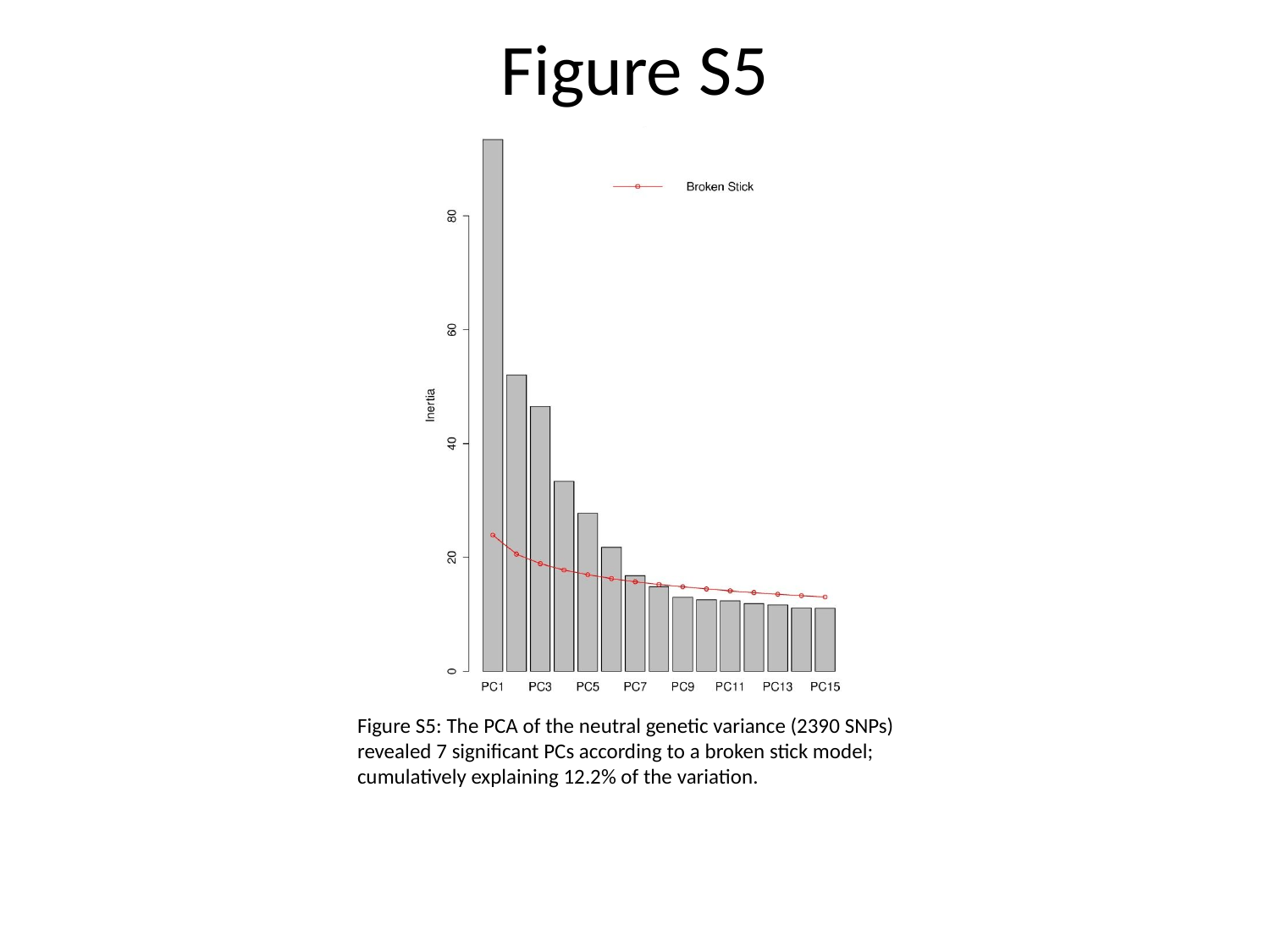

# Figure S5
Figure S5: The PCA of the neutral genetic variance (2390 SNPs) revealed 7 significant PCs according to a broken stick model; cumulatively explaining 12.2% of the variation.

## Slide 7
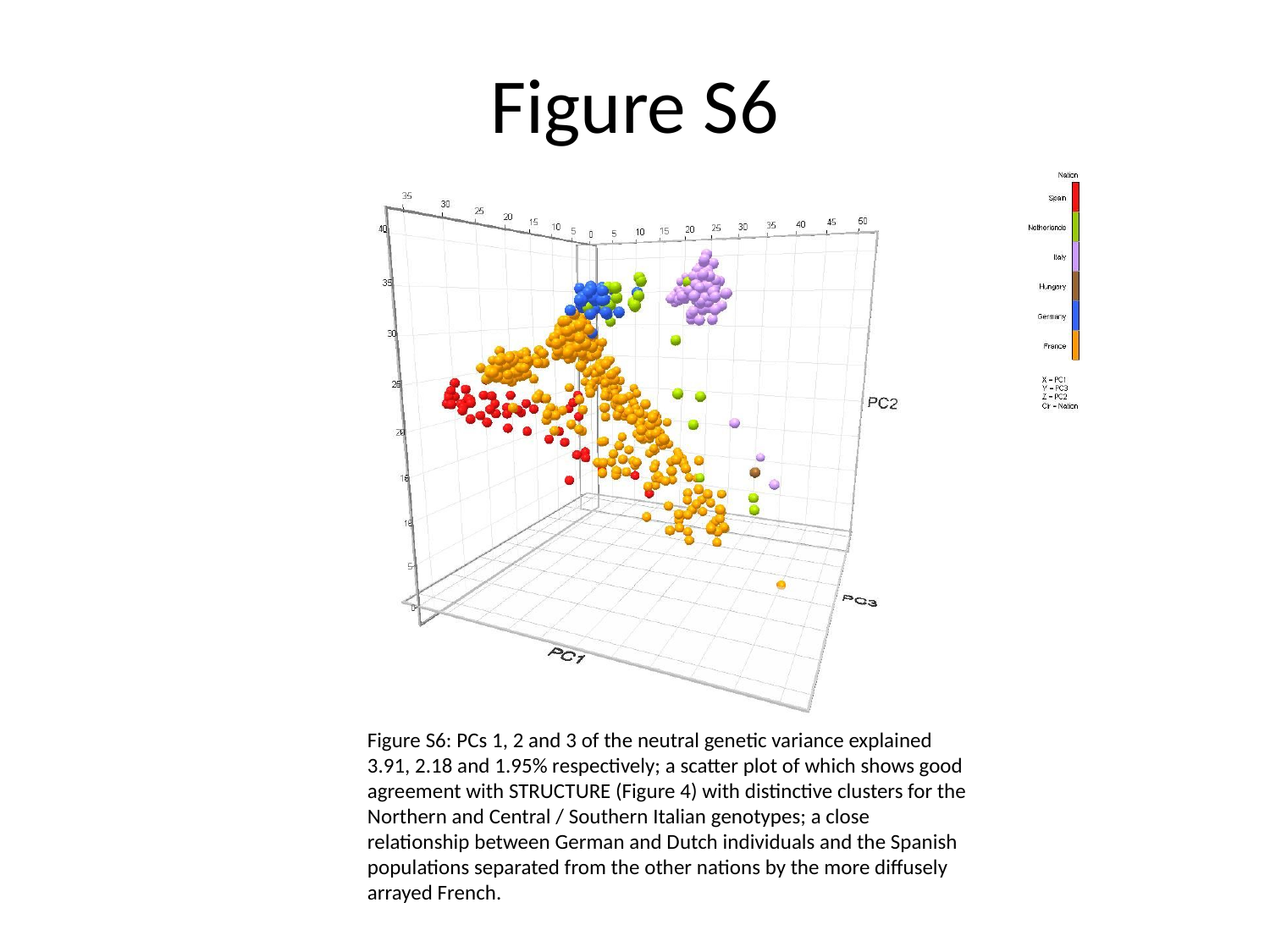

# Figure S6
Figure S6: PCs 1, 2 and 3 of the neutral genetic variance explained 3.91, 2.18 and 1.95% respectively; a scatter plot of which shows good agreement with STRUCTURE (Figure 4) with distinctive clusters for the Northern and Central / Southern Italian genotypes; a close relationship between German and Dutch individuals and the Spanish populations separated from the other nations by the more diffusely arrayed French.

## Slide 8
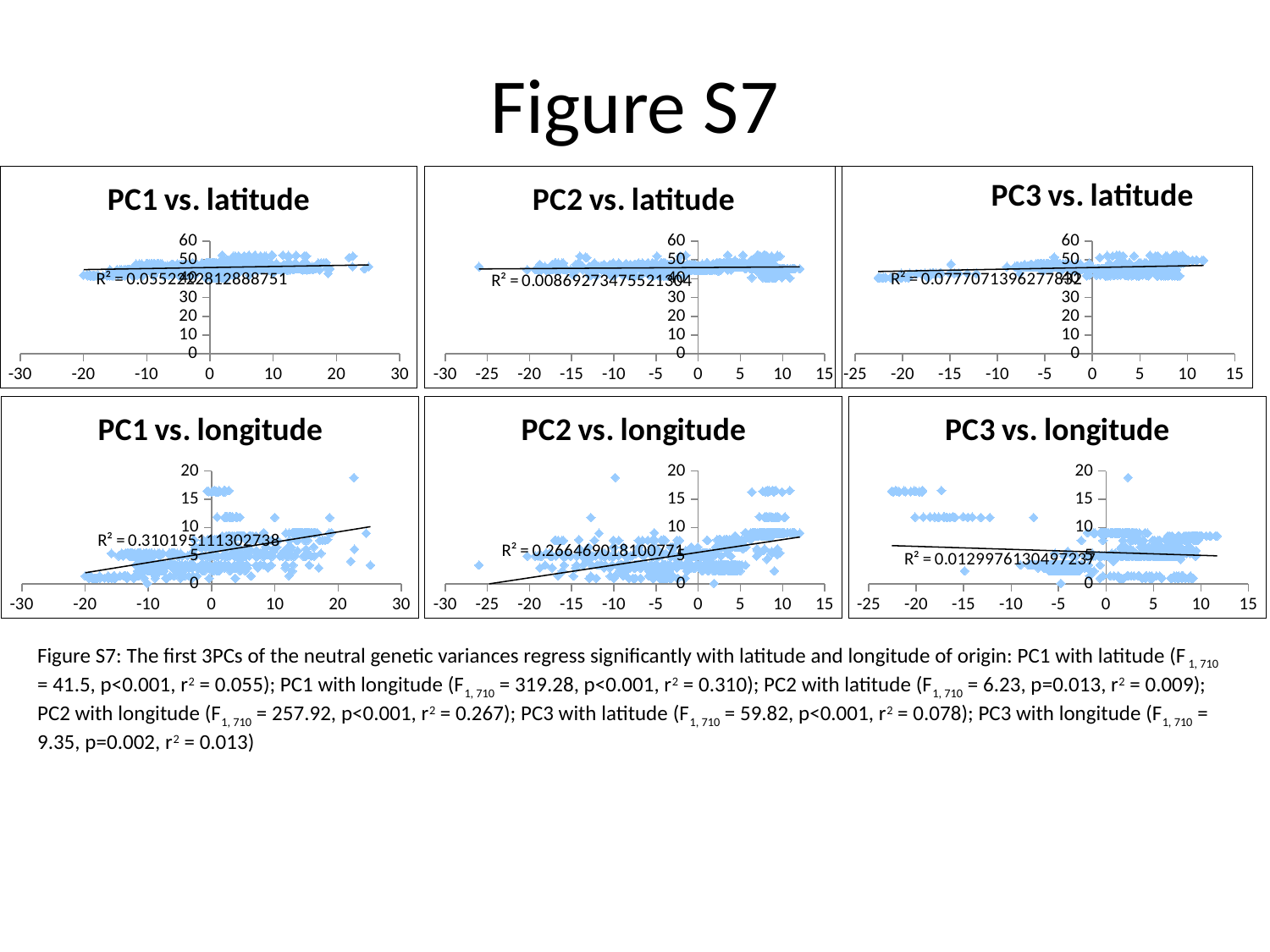

# Figure S7
### Chart: PC1 vs. latitude
| Category | latitude |
|---|---|
### Chart: PC2 vs. latitude
| Category | latitude |
|---|---|
### Chart: PC3 vs. latitude
| Category | latitude |
|---|---|
### Chart: PC1 vs. longitude
| Category | longitude |
|---|---|
### Chart: PC2 vs. longitude
| Category | longitude |
|---|---|
### Chart: PC3 vs. longitude
| Category | longitude |
|---|---|Figure S7: The first 3PCs of the neutral genetic variances regress significantly with latitude and longitude of origin: PC1 with latitude (F1, 710 = 41.5, p<0.001, r2 = 0.055); PC1 with longitude (F1, 710 = 319.28, p<0.001, r2 = 0.310); PC2 with latitude (F1, 710 = 6.23, p=0.013, r2 = 0.009); PC2 with longitude (F1, 710 = 257.92, p<0.001, r2 = 0.267); PC3 with latitude (F1, 710 = 59.82, p<0.001, r2 = 0.078); PC3 with longitude (F1, 710 = 9.35, p=0.002, r2 = 0.013)

## Slide 9
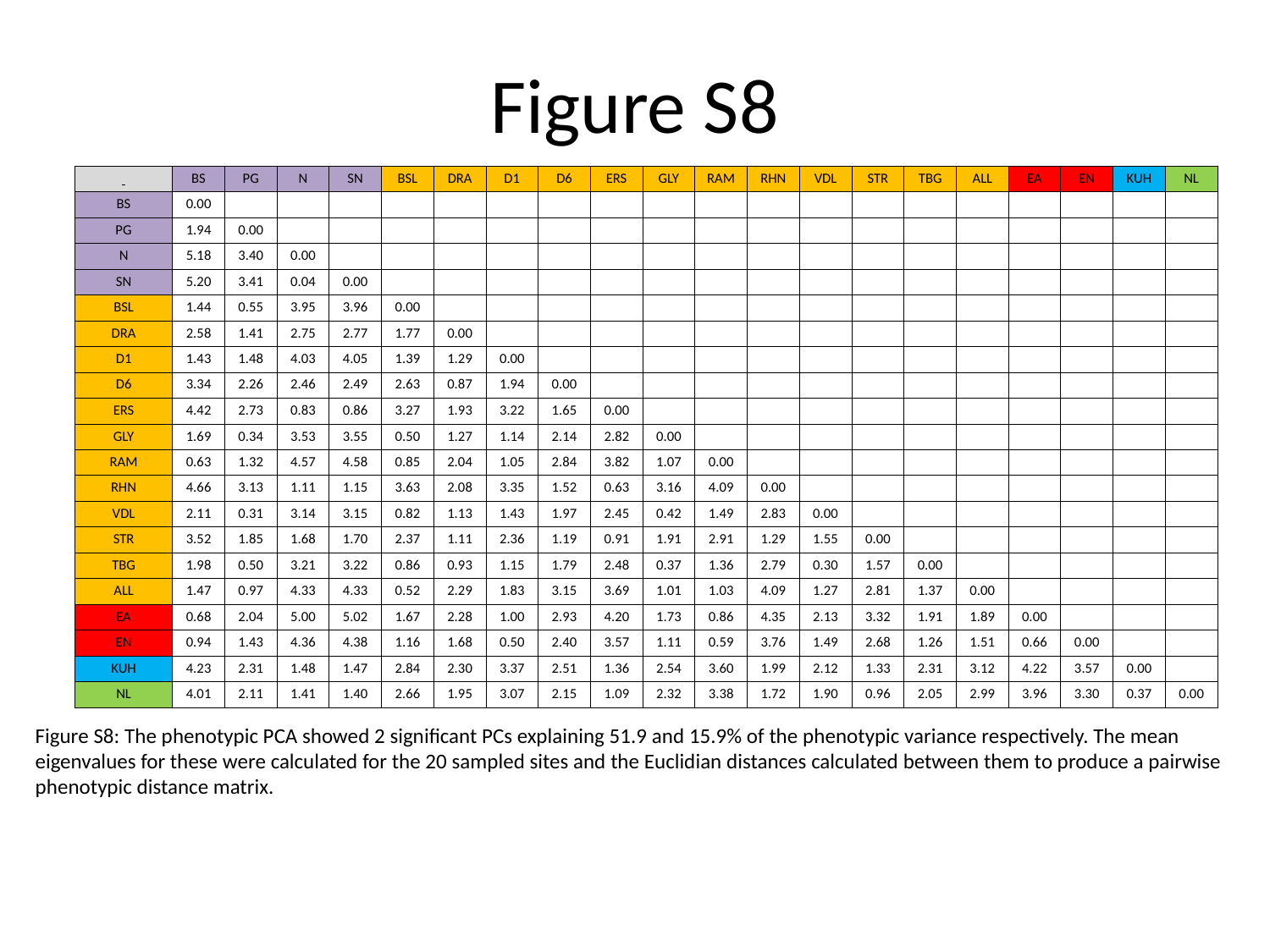

# Figure S8
| | BS | PG | N | SN | BSL | DRA | D1 | D6 | ERS | GLY | RAM | RHN | VDL | STR | TBG | ALL | EA | EN | KUH | NL |
| --- | --- | --- | --- | --- | --- | --- | --- | --- | --- | --- | --- | --- | --- | --- | --- | --- | --- | --- | --- | --- |
| BS | 0.00 | | | | | | | | | | | | | | | | | | | |
| PG | 1.94 | 0.00 | | | | | | | | | | | | | | | | | | |
| N | 5.18 | 3.40 | 0.00 | | | | | | | | | | | | | | | | | |
| SN | 5.20 | 3.41 | 0.04 | 0.00 | | | | | | | | | | | | | | | | |
| BSL | 1.44 | 0.55 | 3.95 | 3.96 | 0.00 | | | | | | | | | | | | | | | |
| DRA | 2.58 | 1.41 | 2.75 | 2.77 | 1.77 | 0.00 | | | | | | | | | | | | | | |
| D1 | 1.43 | 1.48 | 4.03 | 4.05 | 1.39 | 1.29 | 0.00 | | | | | | | | | | | | | |
| D6 | 3.34 | 2.26 | 2.46 | 2.49 | 2.63 | 0.87 | 1.94 | 0.00 | | | | | | | | | | | | |
| ERS | 4.42 | 2.73 | 0.83 | 0.86 | 3.27 | 1.93 | 3.22 | 1.65 | 0.00 | | | | | | | | | | | |
| GLY | 1.69 | 0.34 | 3.53 | 3.55 | 0.50 | 1.27 | 1.14 | 2.14 | 2.82 | 0.00 | | | | | | | | | | |
| RAM | 0.63 | 1.32 | 4.57 | 4.58 | 0.85 | 2.04 | 1.05 | 2.84 | 3.82 | 1.07 | 0.00 | | | | | | | | | |
| RHN | 4.66 | 3.13 | 1.11 | 1.15 | 3.63 | 2.08 | 3.35 | 1.52 | 0.63 | 3.16 | 4.09 | 0.00 | | | | | | | | |
| VDL | 2.11 | 0.31 | 3.14 | 3.15 | 0.82 | 1.13 | 1.43 | 1.97 | 2.45 | 0.42 | 1.49 | 2.83 | 0.00 | | | | | | | |
| STR | 3.52 | 1.85 | 1.68 | 1.70 | 2.37 | 1.11 | 2.36 | 1.19 | 0.91 | 1.91 | 2.91 | 1.29 | 1.55 | 0.00 | | | | | | |
| TBG | 1.98 | 0.50 | 3.21 | 3.22 | 0.86 | 0.93 | 1.15 | 1.79 | 2.48 | 0.37 | 1.36 | 2.79 | 0.30 | 1.57 | 0.00 | | | | | |
| ALL | 1.47 | 0.97 | 4.33 | 4.33 | 0.52 | 2.29 | 1.83 | 3.15 | 3.69 | 1.01 | 1.03 | 4.09 | 1.27 | 2.81 | 1.37 | 0.00 | | | | |
| EA | 0.68 | 2.04 | 5.00 | 5.02 | 1.67 | 2.28 | 1.00 | 2.93 | 4.20 | 1.73 | 0.86 | 4.35 | 2.13 | 3.32 | 1.91 | 1.89 | 0.00 | | | |
| EN | 0.94 | 1.43 | 4.36 | 4.38 | 1.16 | 1.68 | 0.50 | 2.40 | 3.57 | 1.11 | 0.59 | 3.76 | 1.49 | 2.68 | 1.26 | 1.51 | 0.66 | 0.00 | | |
| KUH | 4.23 | 2.31 | 1.48 | 1.47 | 2.84 | 2.30 | 3.37 | 2.51 | 1.36 | 2.54 | 3.60 | 1.99 | 2.12 | 1.33 | 2.31 | 3.12 | 4.22 | 3.57 | 0.00 | |
| NL | 4.01 | 2.11 | 1.41 | 1.40 | 2.66 | 1.95 | 3.07 | 2.15 | 1.09 | 2.32 | 3.38 | 1.72 | 1.90 | 0.96 | 2.05 | 2.99 | 3.96 | 3.30 | 0.37 | 0.00 |
Figure S8: The phenotypic PCA showed 2 significant PCs explaining 51.9 and 15.9% of the phenotypic variance respectively. The mean eigenvalues for these were calculated for the 20 sampled sites and the Euclidian distances calculated between them to produce a pairwise phenotypic distance matrix.

## Slide 10
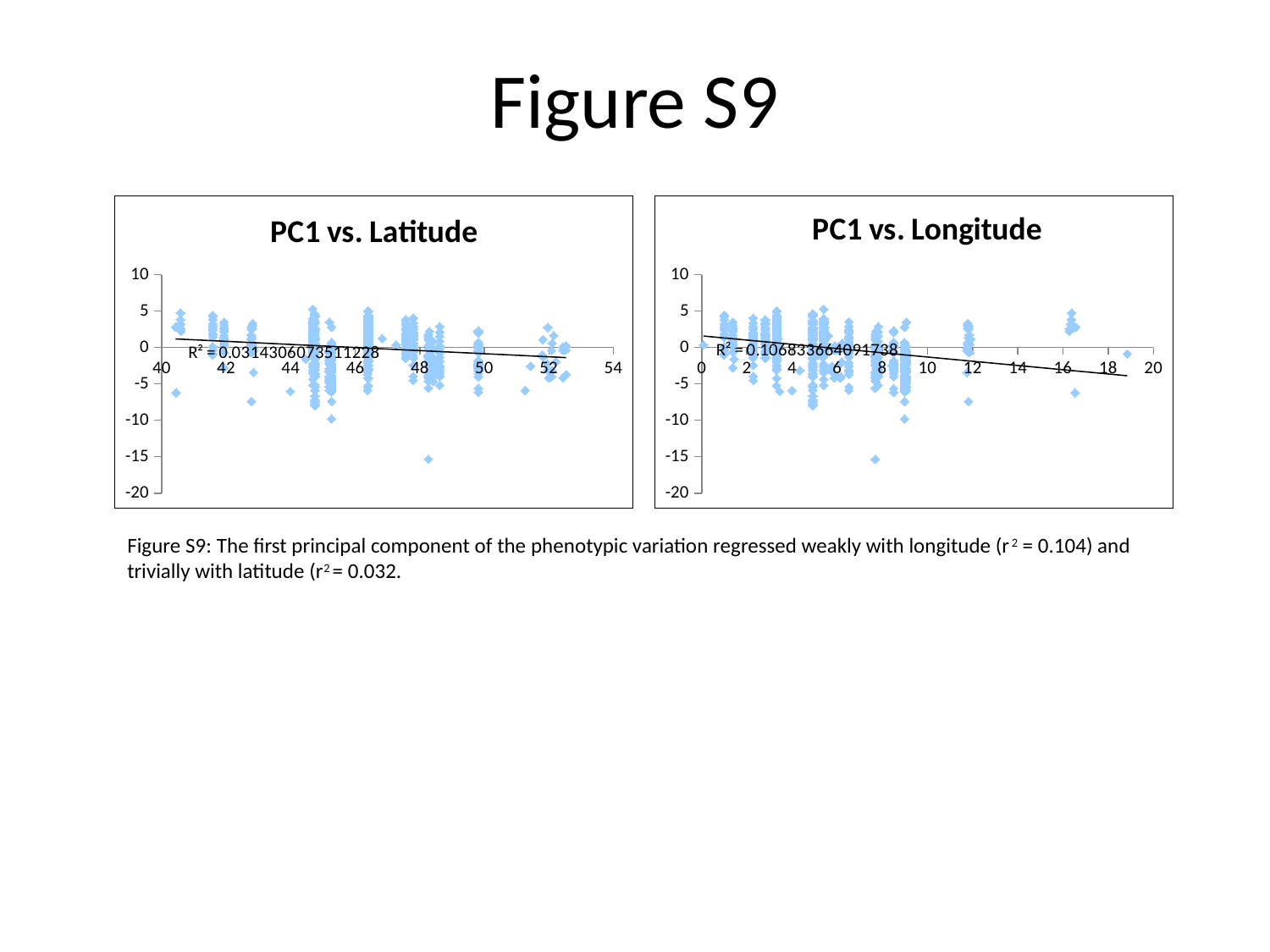

# Figure S9
### Chart: PC1 vs. Latitude
| Category | PC1 |
|---|---|
### Chart: PC1 vs. Longitude
| Category | PC1 |
|---|---|Figure S9: The first principal component of the phenotypic variation regressed weakly with longitude (r2 = 0.104) and trivially with latitude (r2 = 0.032.

## Slide 11
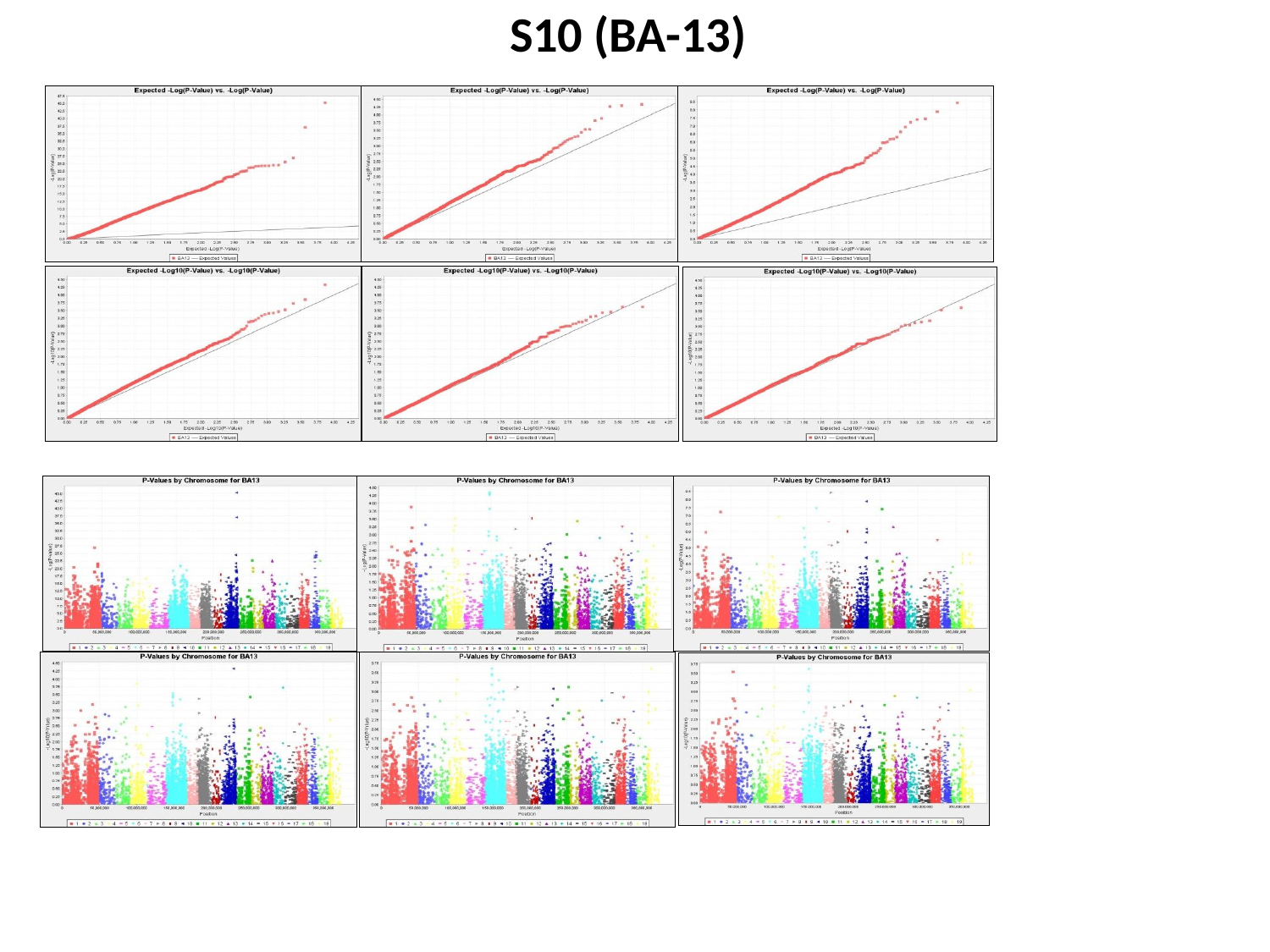

S10 (BA-13)

## Slide 12
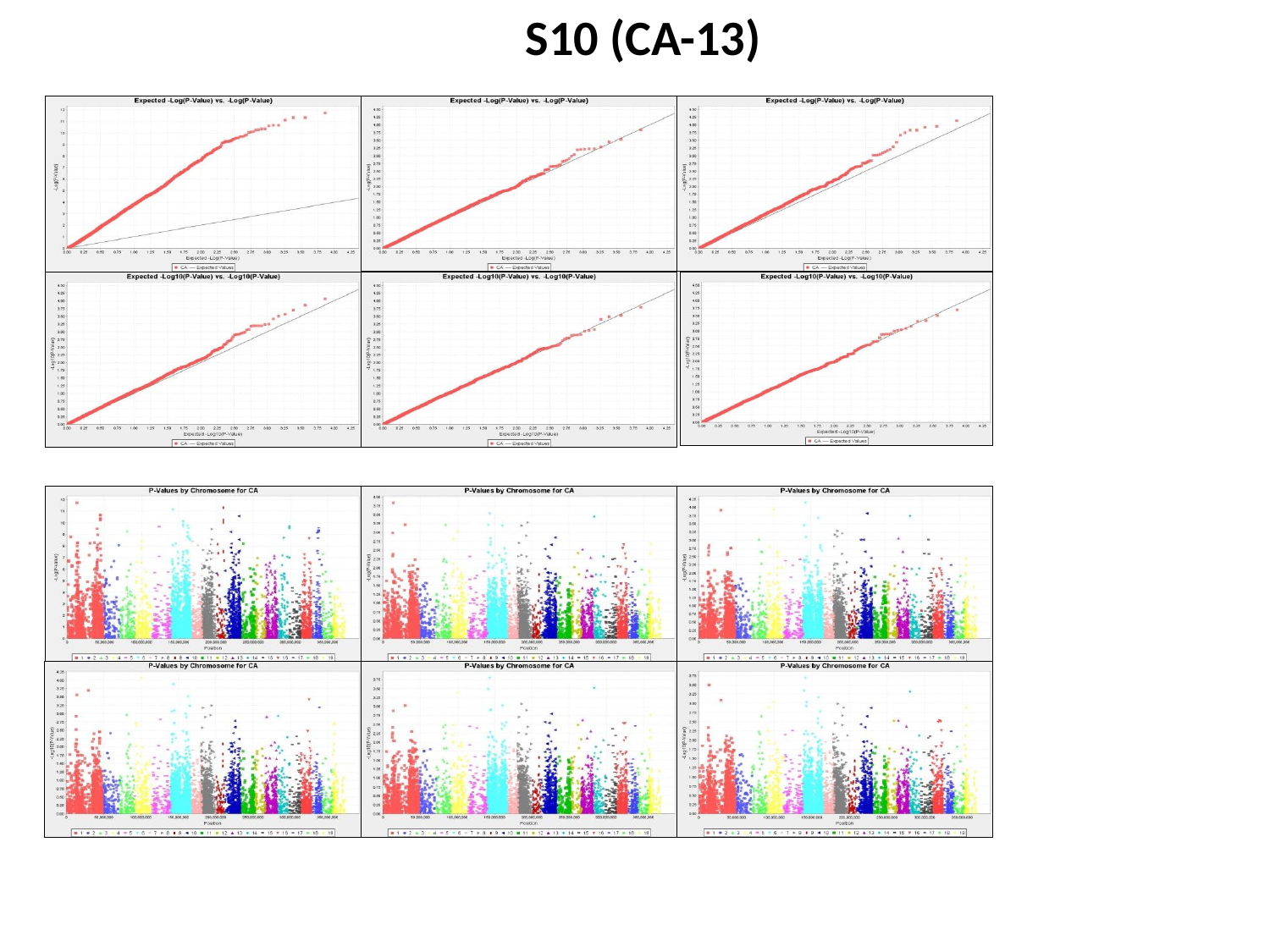

S10 (CA-13)

## Slide 13
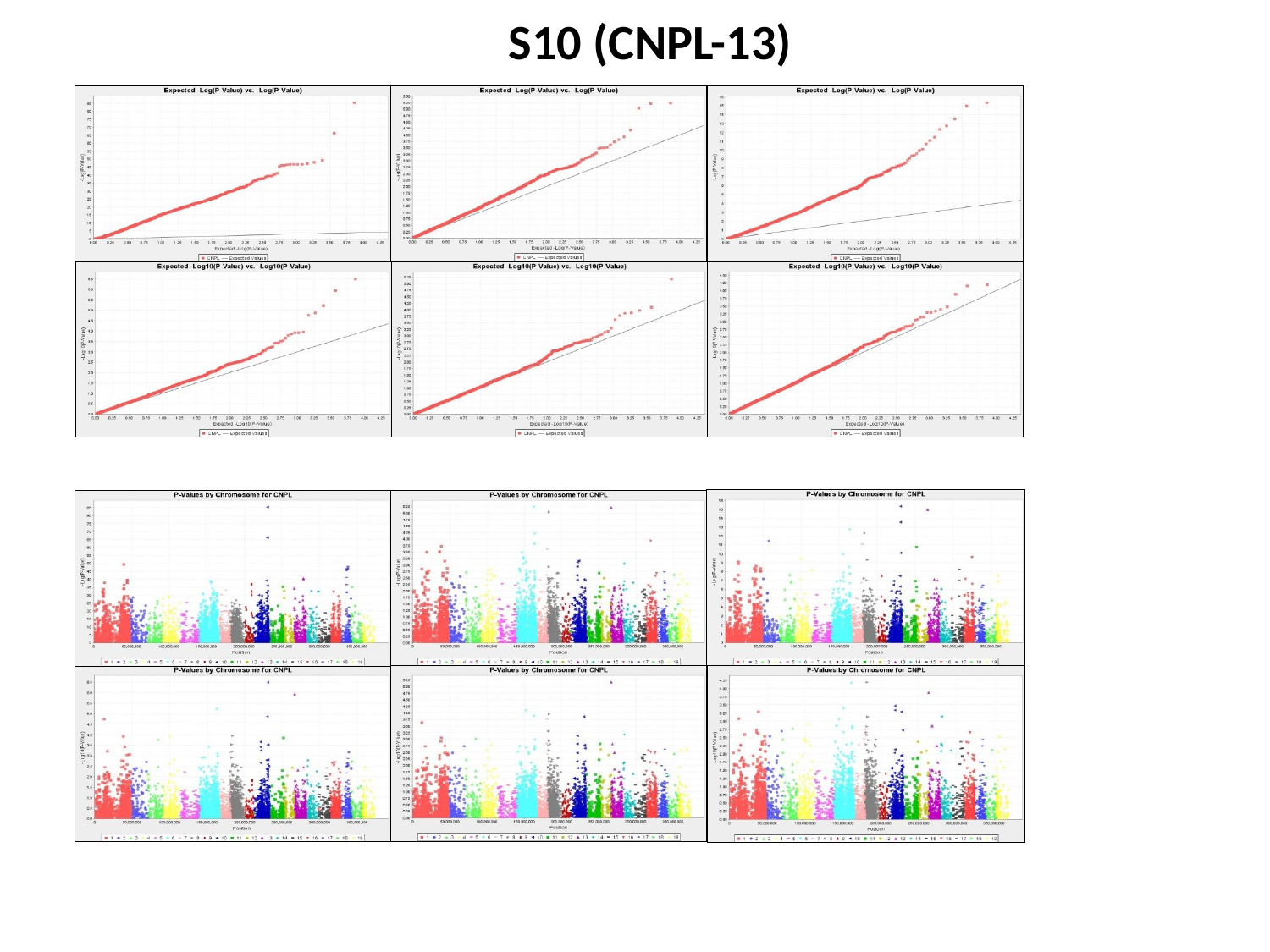

S10 (CNPL-13)

## Slide 14
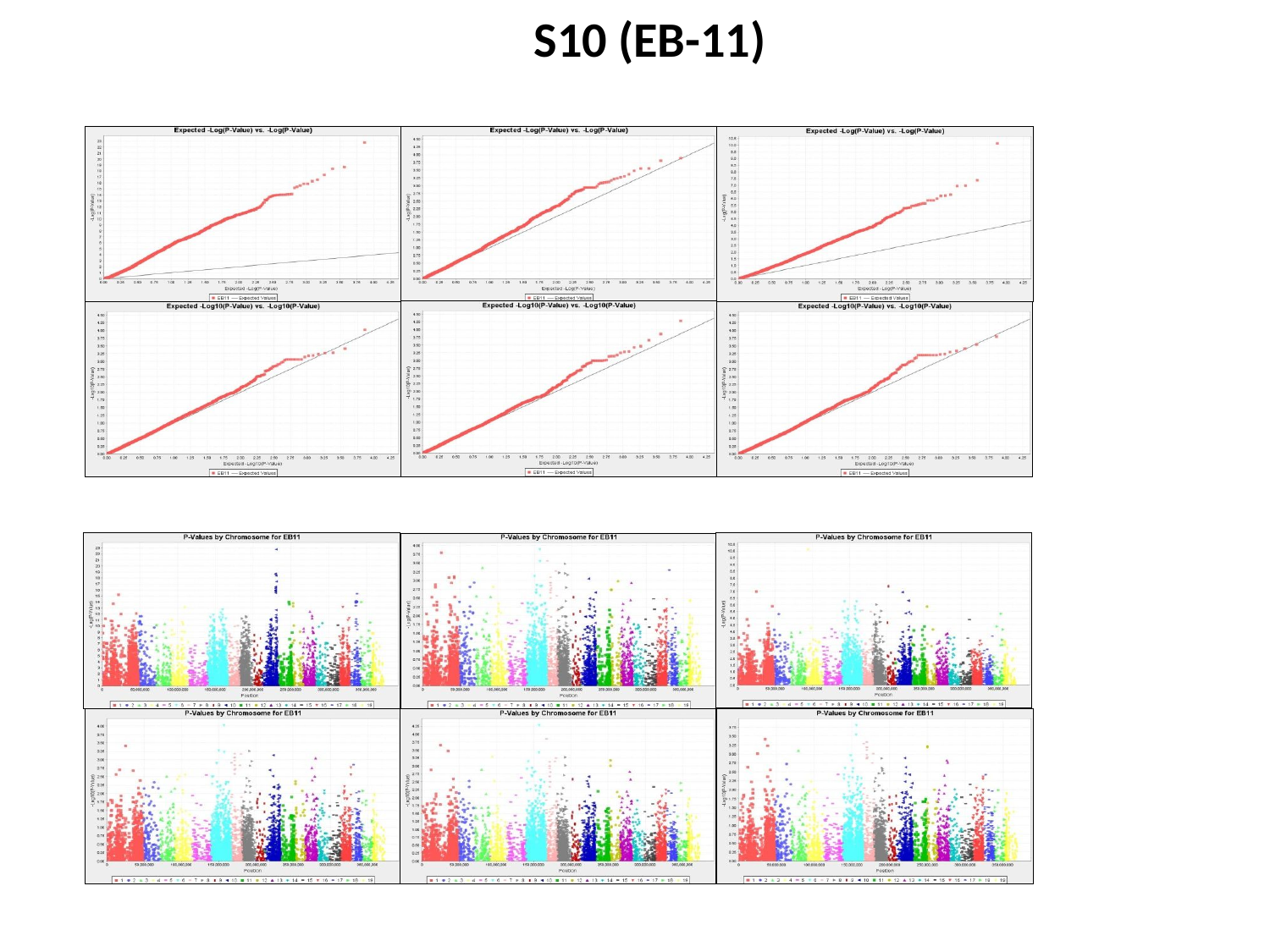

S10 (EB-11)

## Slide 15
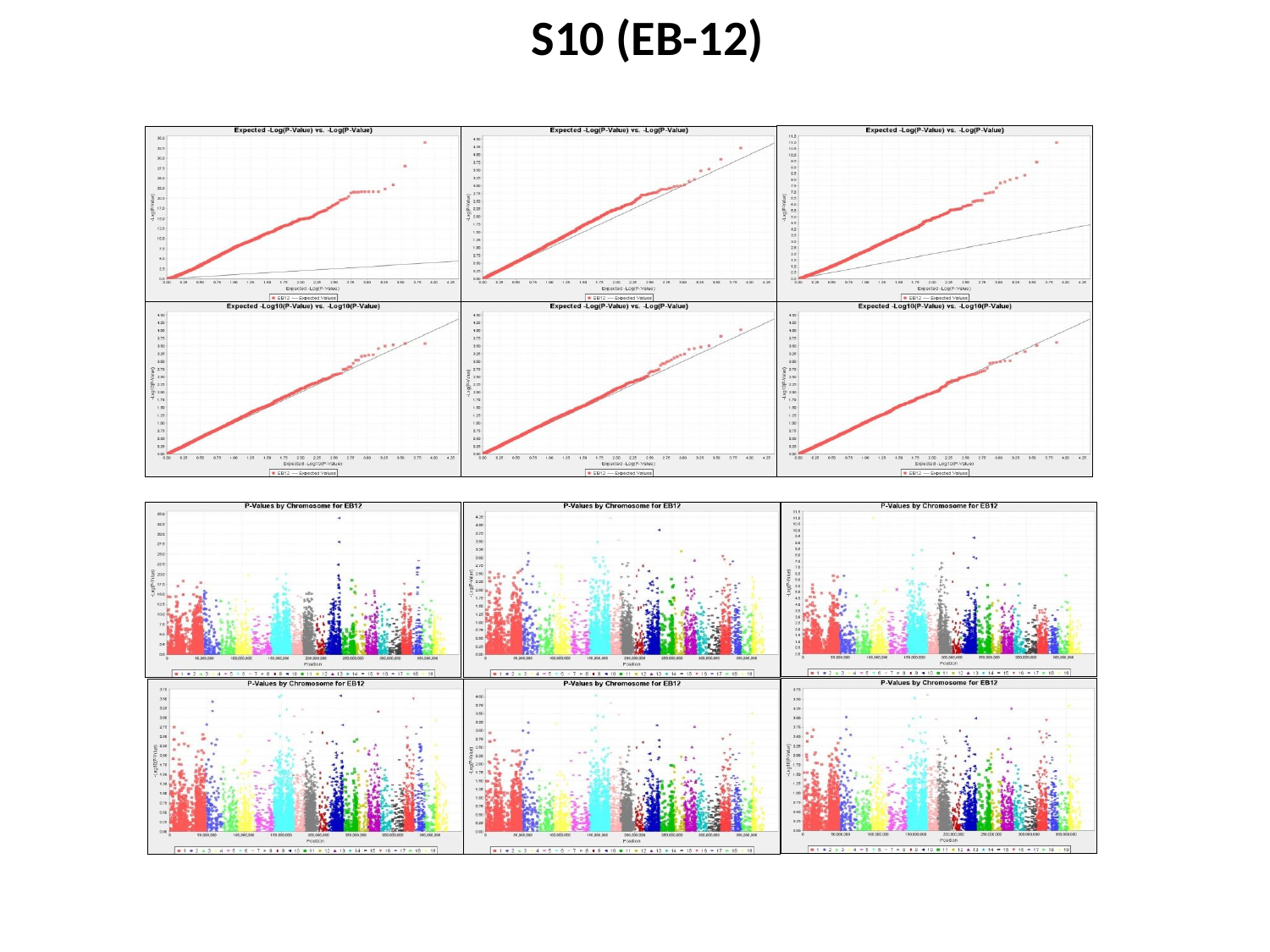

S10 (EB-12)

## Slide 16
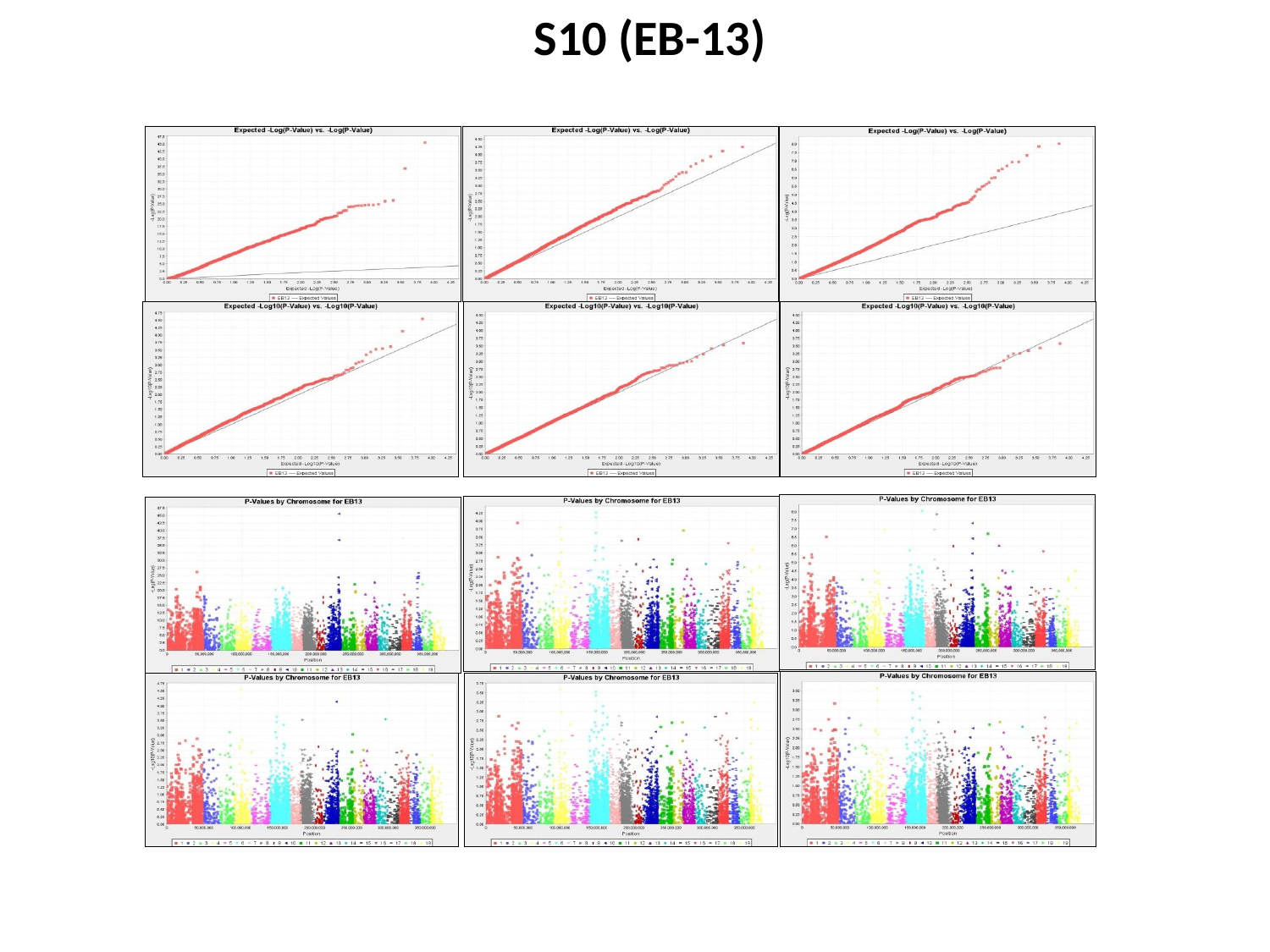

S10 (EB-13)

## Slide 17
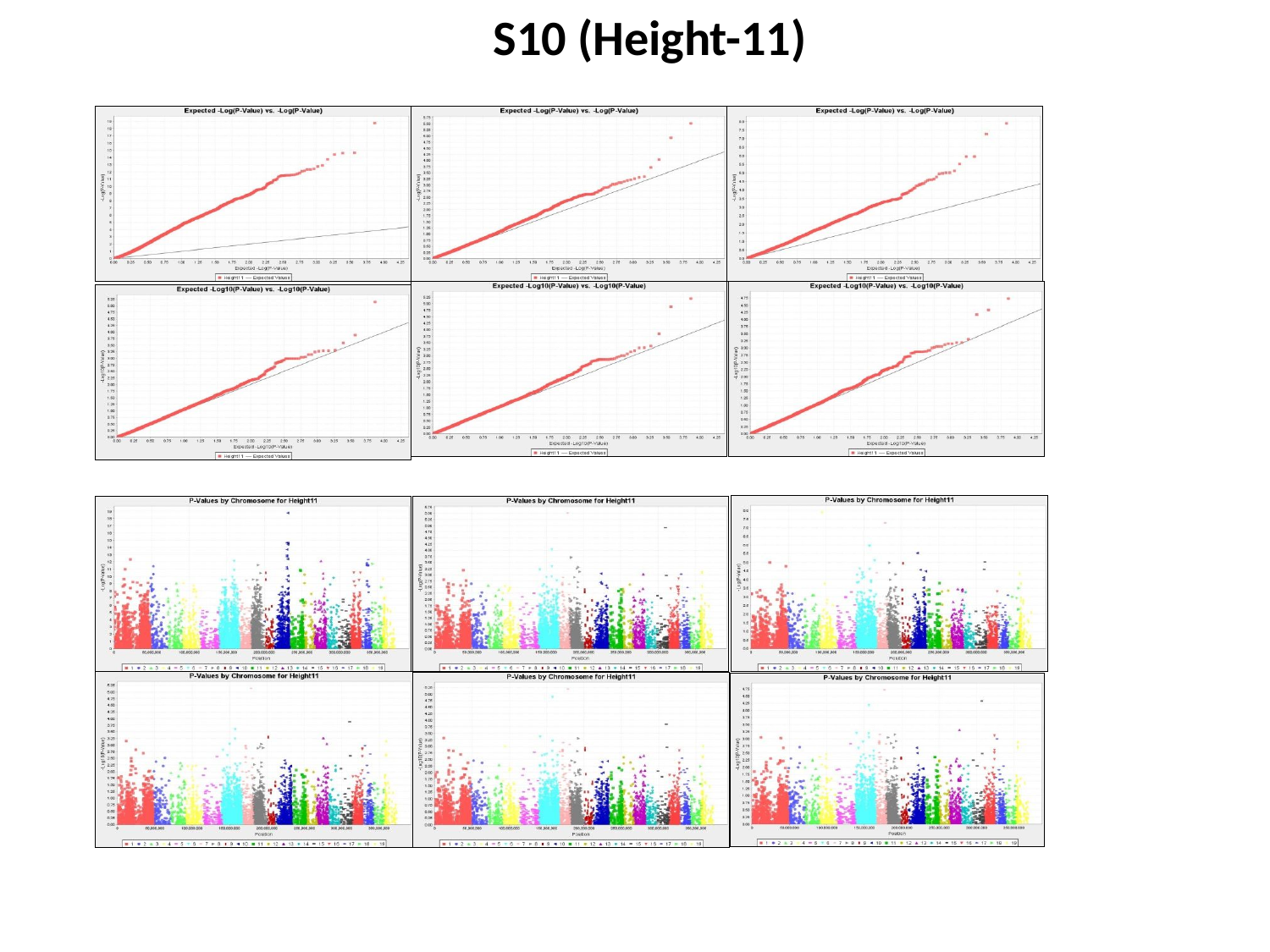

S10 (Height-11)

## Slide 18
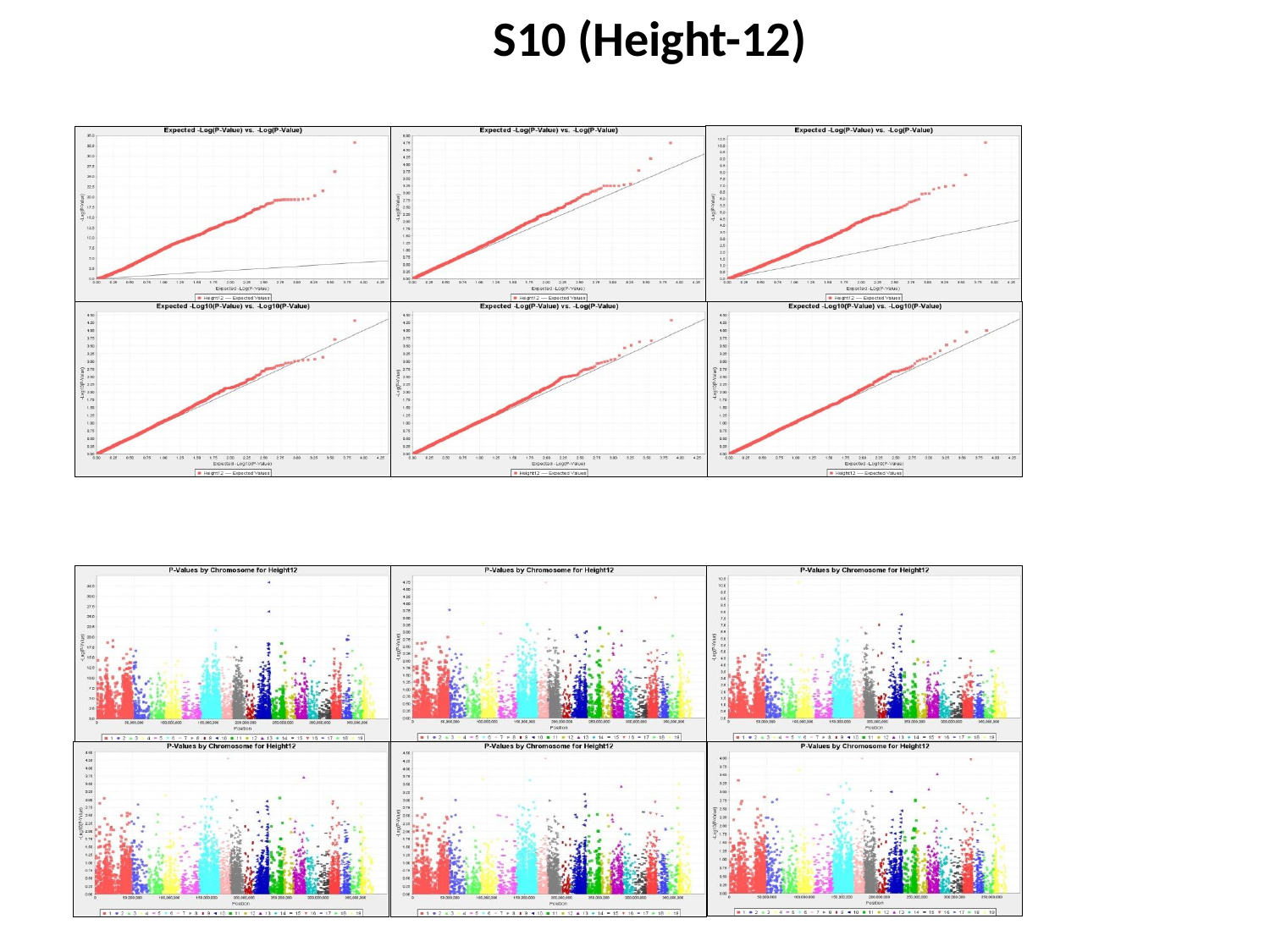

S10 (Height-12)

## Slide 19
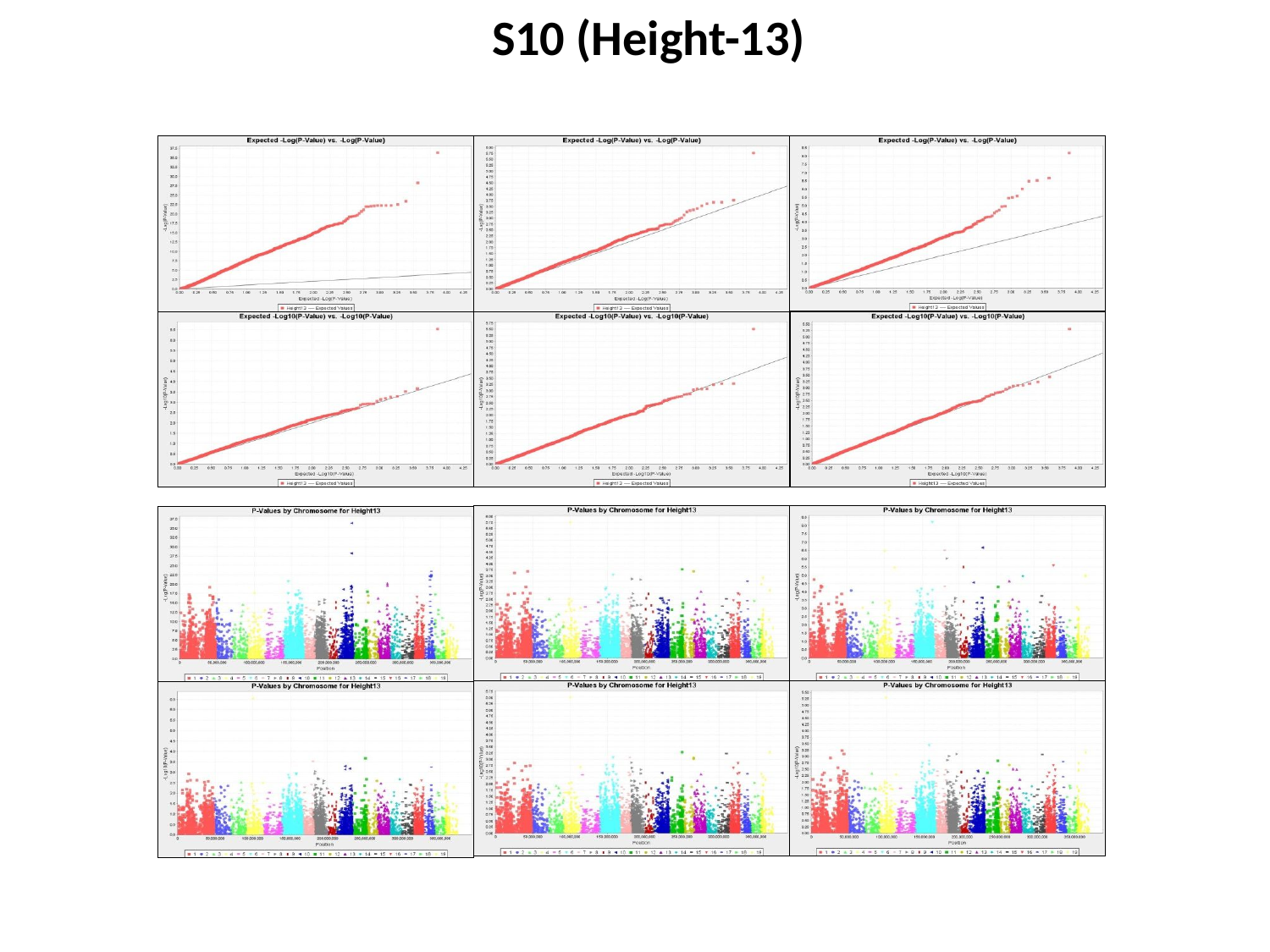

S10 (Height-13)

## Slide 20
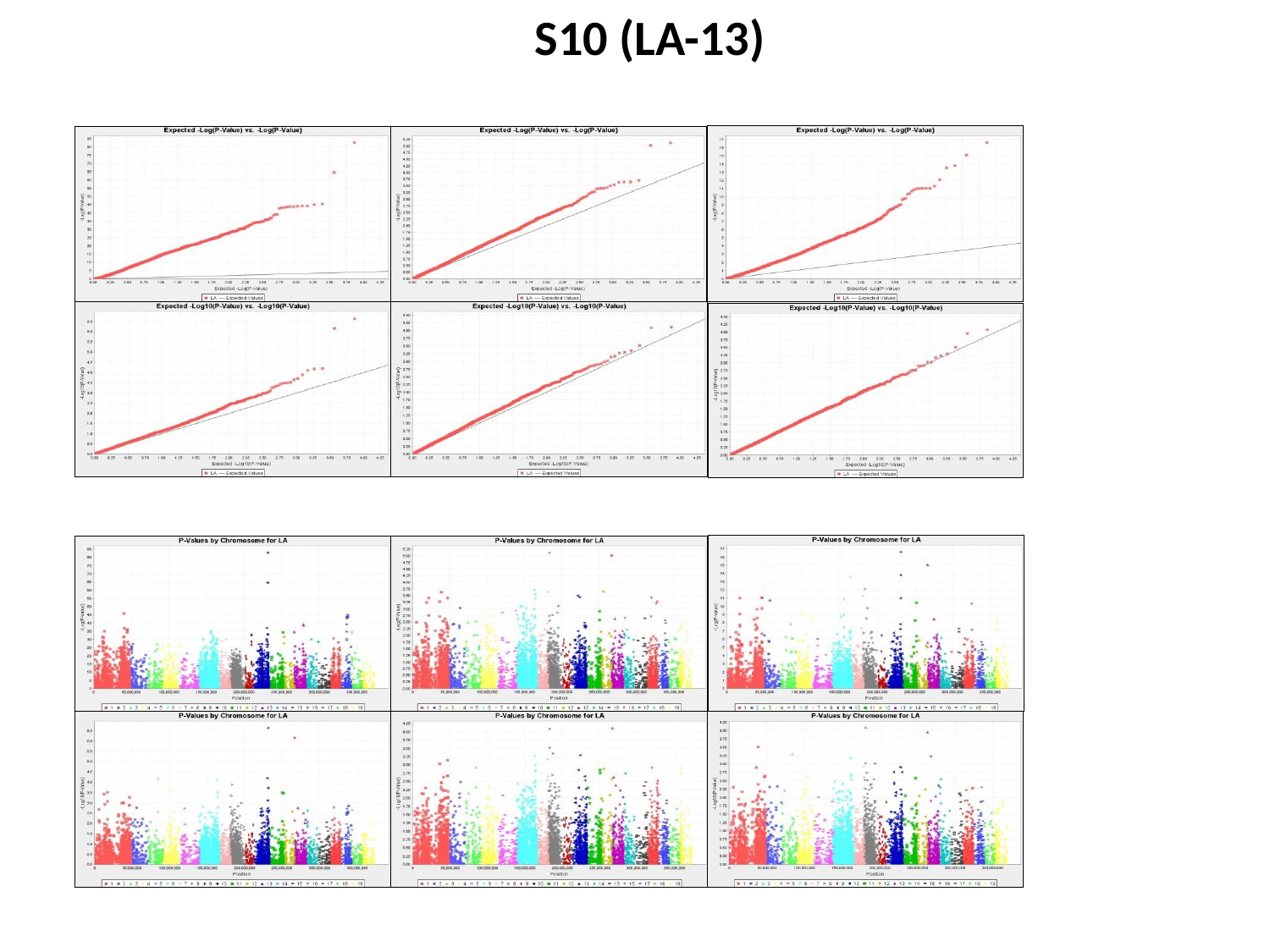

S10 (LA-13)

## Slide 21
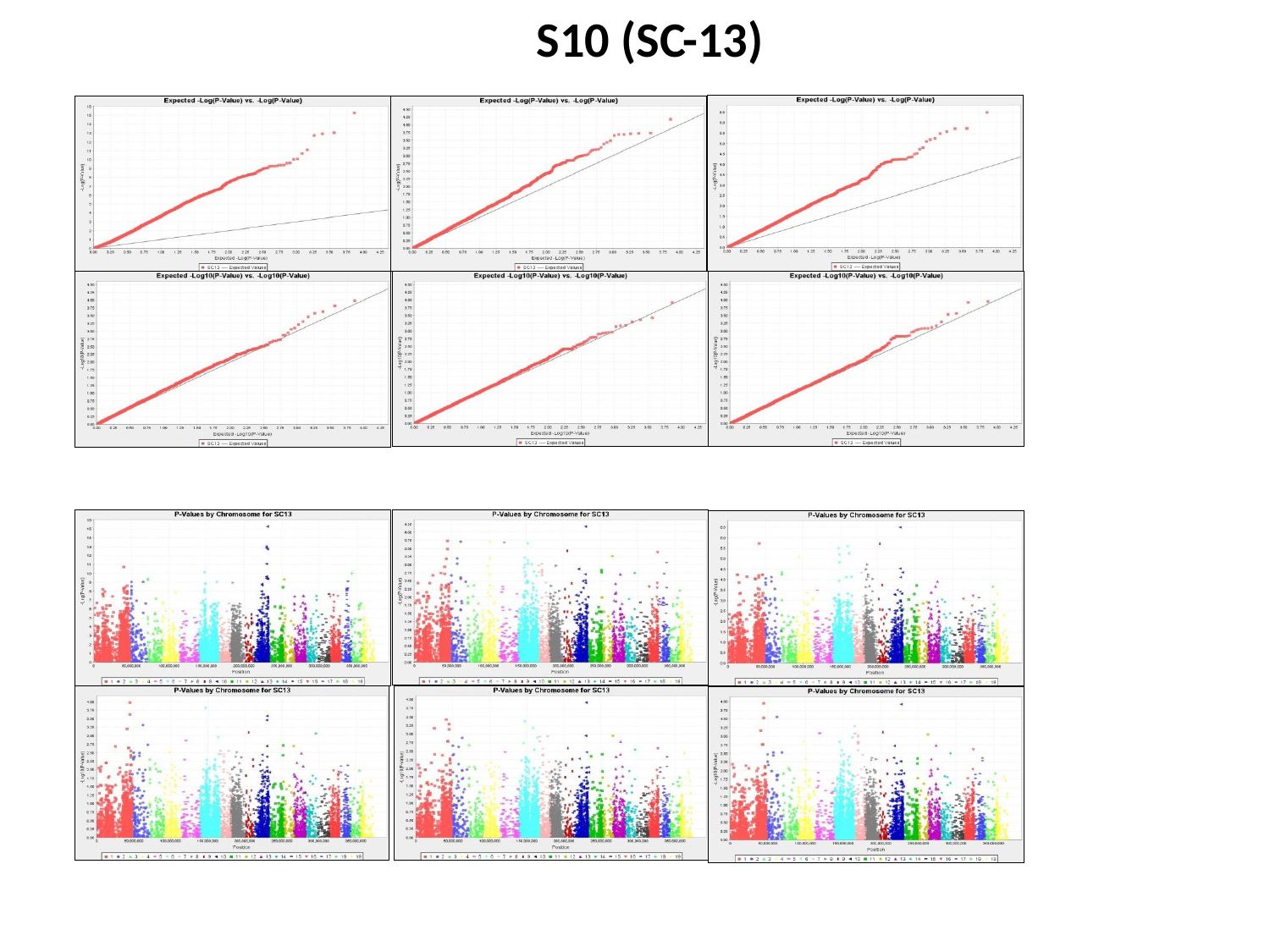

S10 (SC-13)

## Slide 22
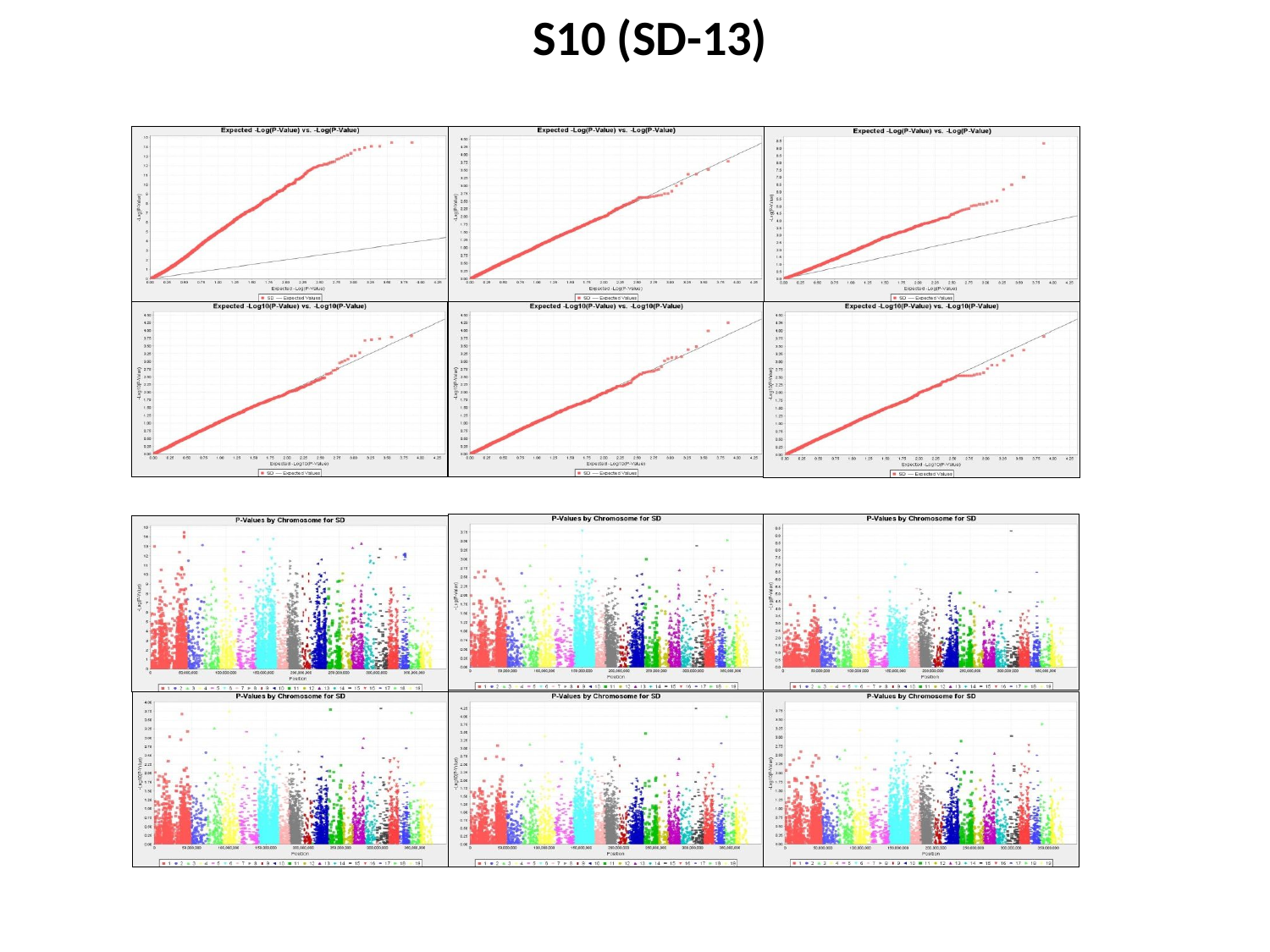

S10 (SD-13)

## Slide 23
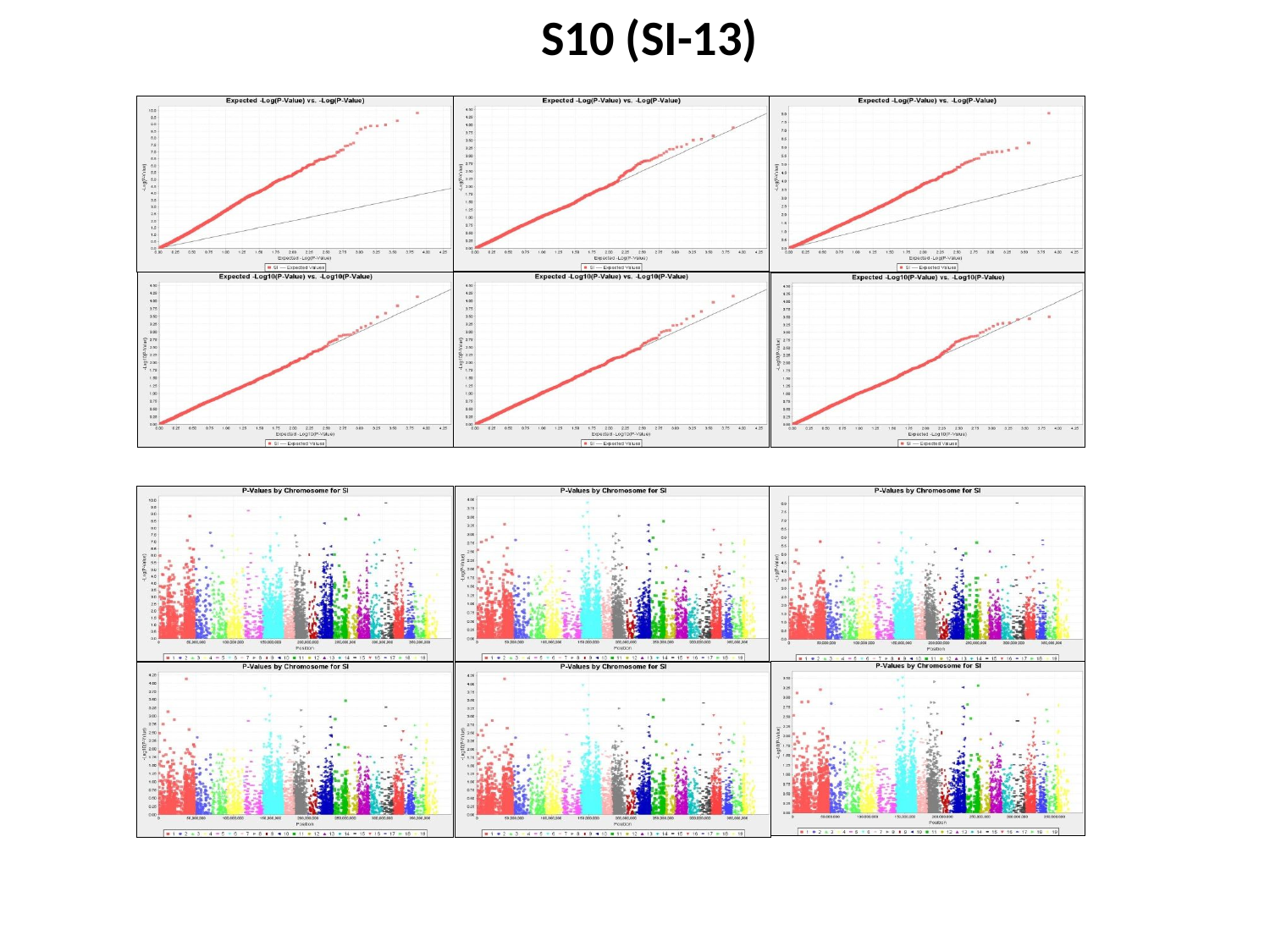

S10 (SI-13)

## Slide 24
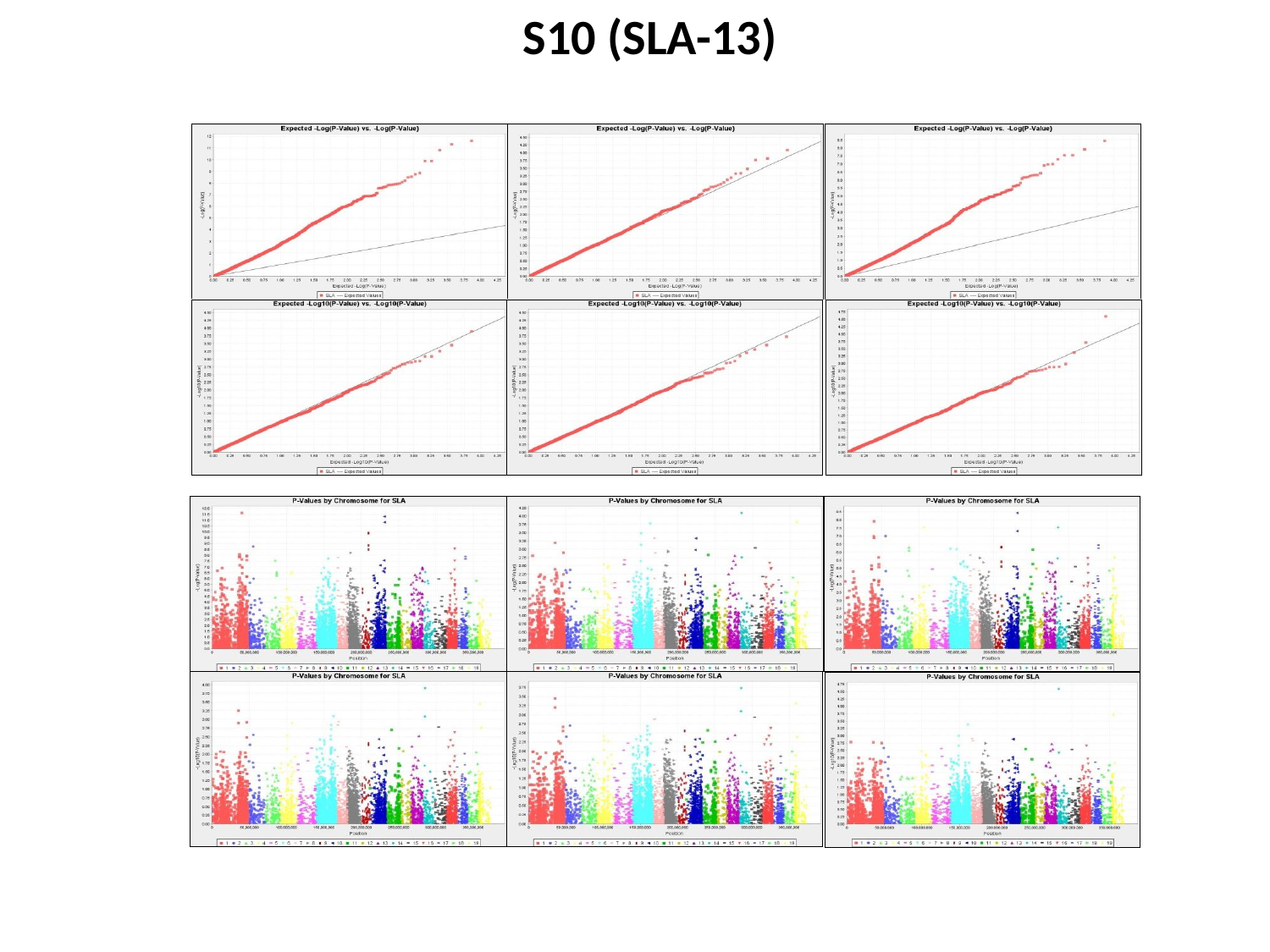

S10 (SLA-13)

## Slide 25
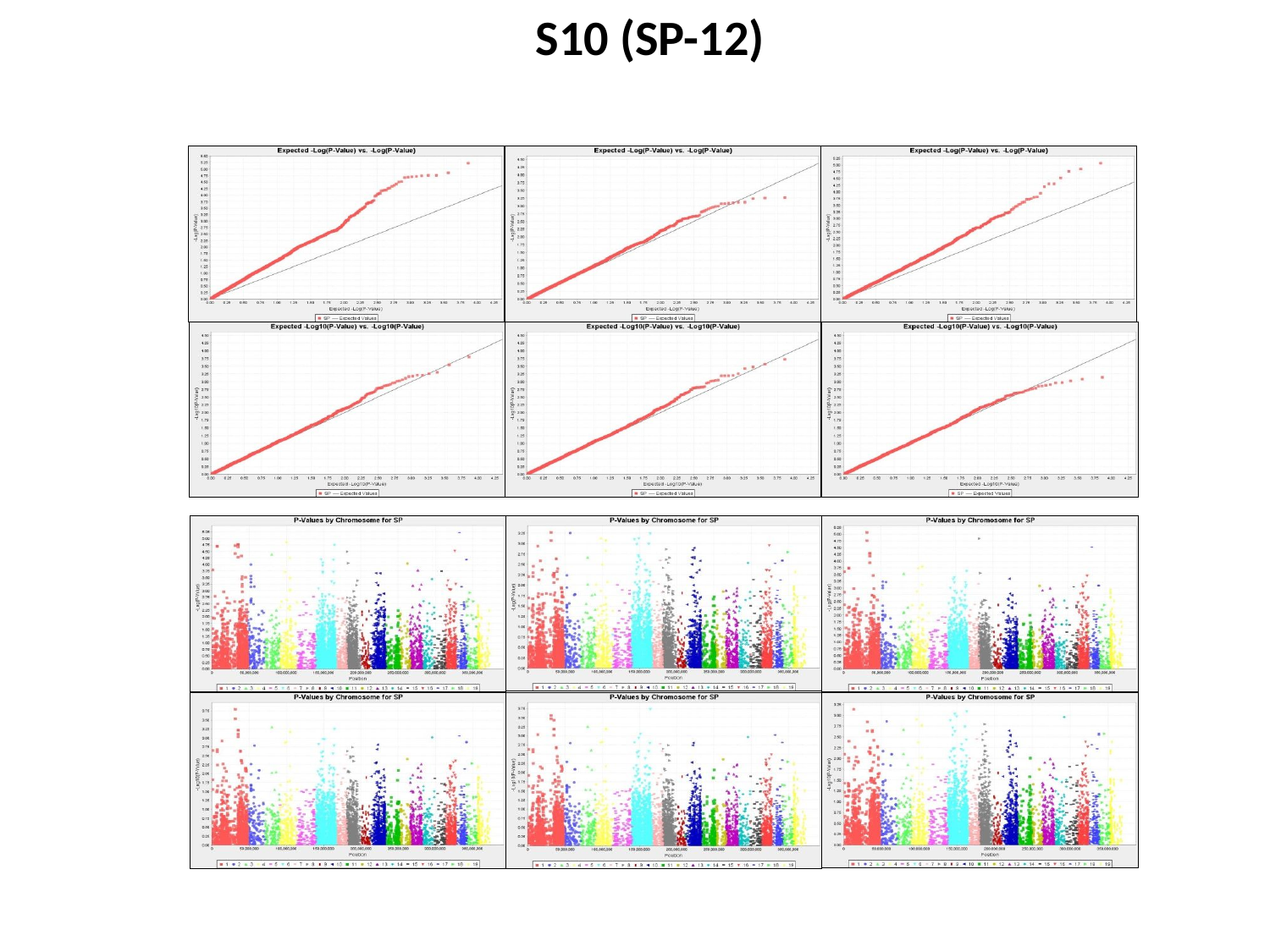

S10 (SP-12)

## Slide 26
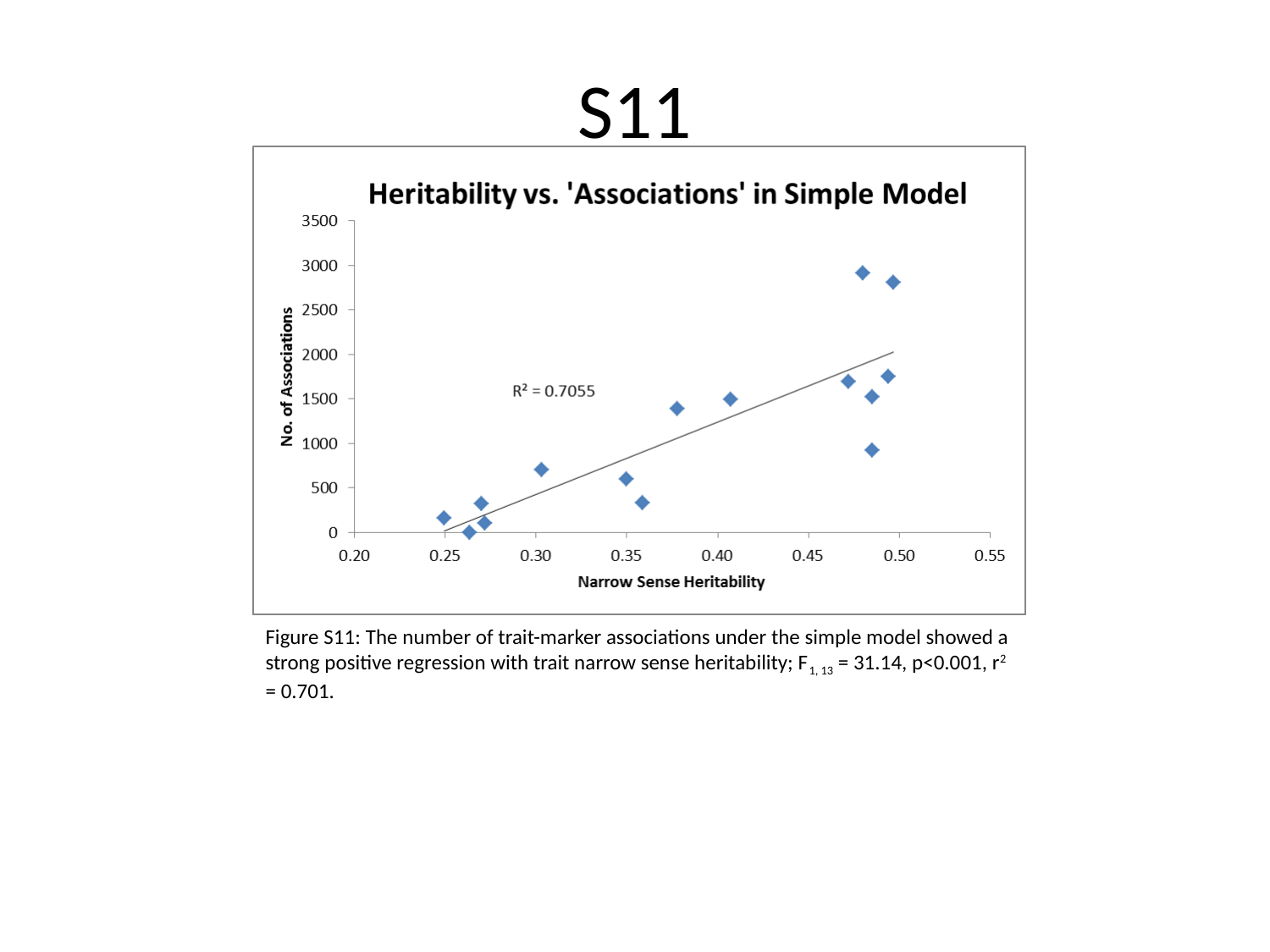

# S11
Figure S11: The number of trait-marker associations under the simple model showed a strong positive regression with trait narrow sense heritability; F1, 13 = 31.14, p<0.001, r2 = 0.701.

## Slide 27
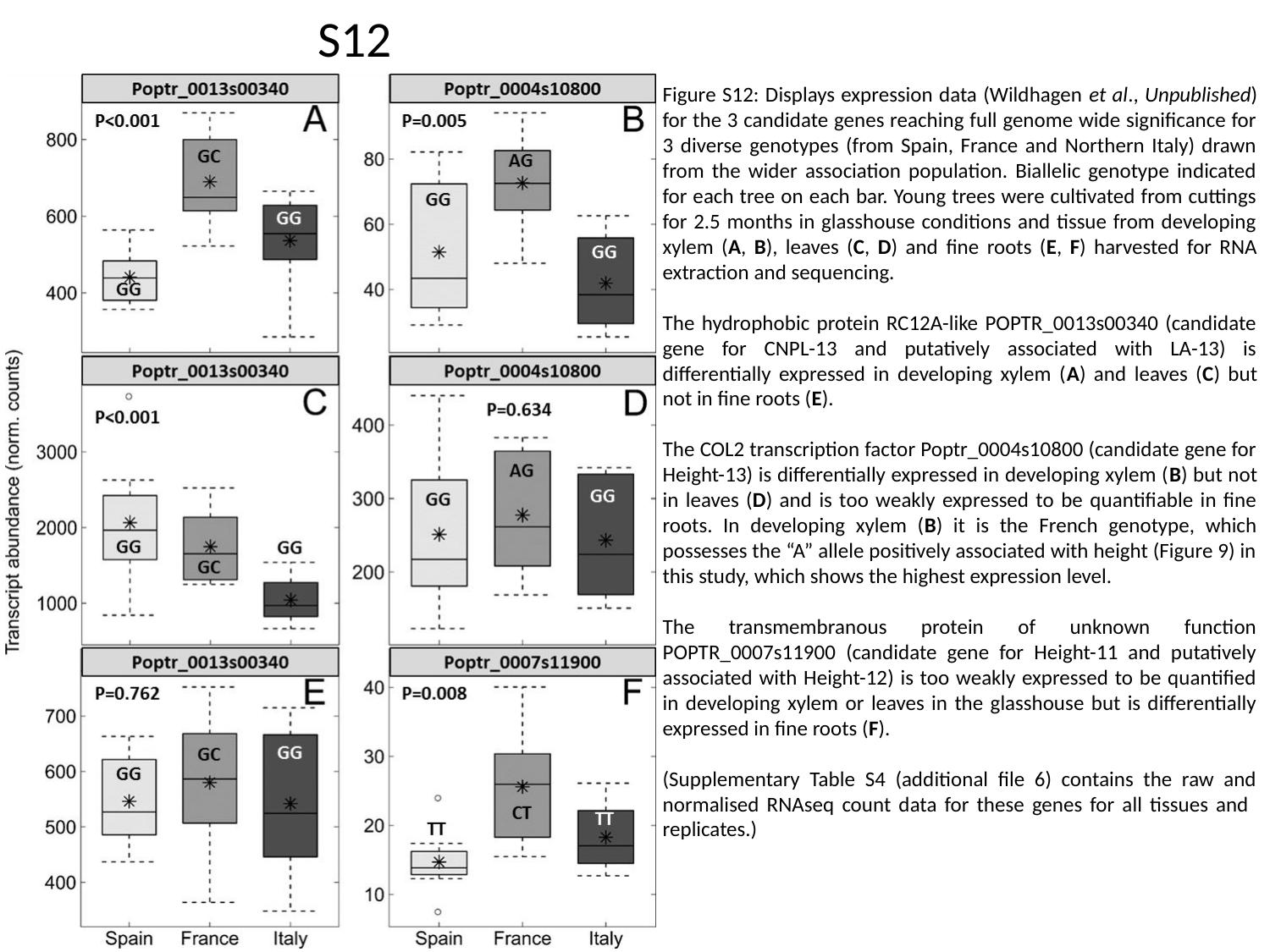

# S12
Figure S12: Displays expression data (Wildhagen et al., Unpublished) for the 3 candidate genes reaching full genome wide significance for 3 diverse genotypes (from Spain, France and Northern Italy) drawn from the wider association population. Biallelic genotype indicated for each tree on each bar. Young trees were cultivated from cuttings for 2.5 months in glasshouse conditions and tissue from developing xylem (A, B), leaves (C, D) and fine roots (E, F) harvested for RNA extraction and sequencing.
The hydrophobic protein RC12A-like POPTR_0013s00340 (candidate gene for CNPL-13 and putatively associated with LA-13) is differentially expressed in developing xylem (A) and leaves (C) but not in fine roots (E).
The COL2 transcription factor Poptr_0004s10800 (candidate gene for Height-13) is differentially expressed in developing xylem (B) but not in leaves (D) and is too weakly expressed to be quantifiable in fine roots. In developing xylem (B) it is the French genotype, which possesses the “A” allele positively associated with height (Figure 9) in this study, which shows the highest expression level.
The transmembranous protein of unknown function POPTR_0007s11900 (candidate gene for Height-11 and putatively associated with Height-12) is too weakly expressed to be quantified in developing xylem or leaves in the glasshouse but is differentially expressed in fine roots (F).
(Supplementary Table S4 (additional file 6) contains the raw and normalised RNAseq count data for these genes for all tissues and replicates.)
GG
GG
TT
