# Supplementary material for: Biomass traits and candidate genes for bioenergy revealed through association genetics in coppiced European Populus nigra (L.)
Source: Biotechnol Biofuels. 2016 Sep 8;9(1):195. doi: 10.1186/s13068-016-0603-1 (PMC5017058; doi:10.1186/s13068-016-0603-1)
Supplement: Supplementary file 3 — 10.1186/s13068-016-0603-1 containing supplementary table S1; showing the results of the general linear model run for all phenotypic traits. [file 13068_2016_603_MOESM3_ESM.docx]

| Trait | Abb. | Site of Origin | Genotype | Block | Run |
| --- | --- | --- | --- | --- | --- |
| Total Basal Stem Area 2013 | BA-13 | *** | *** | *** | - |
| Leaf Cell Area 2013 | CA-13 | *** | *** | *** | - |
| Epidermal Cell No. Per Leaf 2013 | CNPL-13 | *** | *** | *** | - |
| Estimated Biomass in 2011 | EB-11 | *** | *** | *** | - |
| Height of tallest stem 2011 | Height-11 | *** | *** | *** | - |
| Estimated Biomass 2012 | EB-12 | *** | *** | *** | - |
| Height 12 | Height-12 | *** | *** | *** | - |
| Estimated Biomass 13 | EB-13 | *** | *** | *** | - |
| Height 13 | Height-13 | *** | *** | *** | - |
| Estimated Biomass 13 | EB-13 | *** | *** | *** | - |
| Leaf Area (size of first mature leaf) 13 | LA-13 | *** | *** | *** | - |
| Stomatal Density 13 | SD-13 | *** | *** | *** | - |
| Stomatal Index 13 | SI-13 | *** | *** | *** | - |
| Specific Leaf Area 13 | SLA-13 | *** | *** | *** | - |
| Saccharification Potential 12 | SP-12 | 0.056 | *** | *** | *** |

Table S1: Results (p-values) for Northington bioenergy trait data analysis according to the general linear model: Y_ijk_ = µ + S_i_ + G_j(i)_ + B_k_ + ε_ijk_ where µ is the group mean, S_i_ is the effect of site of origin considered as fixed and G_j(i)_ and B_k_ are the effects of genotype (nested within site) and block respectively both considered as random. In the case of saccharification potential the factor ‘Run’ was additionally included as a random effect. Only the site effect for SP-12 failed to reach significance at p<0.001 (***).
